# Functional characterization of CYP96T1-like cytochrome P450 from *Lycoris aurea* catalyzing *para-para*′ and *para-ortho*′ oxidative coupling in Amaryllidaceae alkaloids biosynthesis

**DOI:** 10.3389/fpls.2024.1438102

**Published:** 2024-10-02

**Authors:** Zhengtai Liu, Bin Sun, Junde Li, Yiyu Xiang, Rong Wang, Xiaoran Jiang, Xinning Zhu, Sheng Xu, Ren Wang

**Affiliations:** ^1^ Nanjing University of Chinese Medicine, Nanjing, China; ^2^ Institute of Botany, Jiangsu Province and Chinese Academy of Sciences, Nanjing, China; ^3^ Jiangsu Key Laboratory for the Research and Utilization of Plant Resources, Nanjing, China

**Keywords:** *Lycoris aurea*, full-length transcriptome, Amaryllidaceae alkaloids, C-C oxidative coupling, cytochrome P450

## Abstract

Amaryllidaceae alkaloids (AAs) are complex plant secondary metabolites possessing a wide range of biological activities. 4′-O-methylnorbelladine (4OMN) is the branchpoint intermediate for the entire AAs, and was the last common intermediate before AA pathway branches diverge. The cyclization of 4OMN by C-C oxidative coupling, which can afford *para-para*′, *ortho-para*′, and *para-ortho*′ scaffold, was catalyzed by cytochrome P450 96T (CYP96T) family enzymes. To clarify the mechanisms involved in this controversial step, four CYP96T homologs (LauCYP96T1, LauCYP96T1-like-1, LauCYP96T1-like-2 and LauCYP96T1-like-3) were cloned from the full-length transcriptome of *Lycoris aurea*. All the four LauCYP96T are localized to endoplasmic reticulum. Functional analysis reveals that LauCYP96T1 and LauCYP96T1-like proteins display inverted regioselectivity for oxidative coupling of 4OMN, in which LauCYP96T1 and LauCYP96T1-like-2 dominantly afford *para-para*′ scaffold, and LauCYP96T1-like-1 and LauCYP96T1-like-3 are responsible for *para-ortho*′ scaffold formation. Using molecular homology modeling and docking studies, we predicted models for the binding of 4OMN to LauCYP96T, and identified two amino acid residues that might be responsible for the dominant changes in generated products of *para-ortho*′ and *para-para*′ oxidative coupling. Our results highlight the functional diversity and promiscuity of LauCYP96T enzymes and might provide valuable information for Amaryllidaceae alkaloid production.

## 1 Introduction

Plants produce a wide variety of compounds that are classified into primary and secondary metabolites. The secondary metabolites, also known as specialized metabolites or natural products, usually play pivotal roles in helping plants adapt to the environment (Wang et al., 2022). In addition, plant secondary metabolites are the sources of medicines, drugs, and flavors, with beneficial effects for human’s daily life (Chiocchio et al., 2021). For medicinal plants, the abundance of secondary metabolites such as alkaloids, phenylpropanoids, and terpenoids makes them rich in nutraceutical and pharmaceutical properties (Zhao et al., 2023).

*Lycoris aurea* is an important medicinal belonging to Amaryllidaceae family. It produces the Amaryllidaceae-type alkaloids with great therapeutic promise, including immunostimulatory, anti-malarial, anticancer, and antiviral properties (Jin and Yao, 2019). For example, galantamine has been licensed for the treatment of Alzheimer’s disease because of its reversible inhibitor activity of acetylcholinesterase (Mucke, 2015). Galantamine is usually isolated from Amaryllidaceae plants such as daffodil, snowdrop, and snowflake (Marco-Contelles et al., 2006). However, due to the low quantities of galantamine in nature, extraction from plants would pose a threat to their habitats and to the growth of native plants (Berkov et al., 2009). In addition, because of the complex structure, galantamine’s synthesis is still challenging (Cheng et al., 2024).

Synthetic biology provides a promising alternative for the cost-effective and sustainable production of plant natural products (Liu et al., 2023). Many industrially important plant secondary metabolites have been produced by engineering microbes. For example, semisynthetic artemisinin could be produced at industrial scale based on yeast fermentation (Paddon et al., 2013). Recently, some long plant biosynthesis pathways of tropane alkaloids, monoterpene indole alkaloids, and vaccine adjuvant Q-21 have been reconstituted into microbial hosts for production of these secondary metabolites (Srinivasan and Smolke, 2020; Zhang et al., 2022; Liu et al., 2024). In addition to microbes, plants also represent a unique and powerful chassis for synthetic biology, and various secondary metabolites including alkaloids, terpenoids, polyketides, and glucosinolates, have been successfully produced via transient coexpression in *Nicotiana benthamiana* and other plant species (Liu et al., 2023). Since the complete biosynthetic pathway for Amaryllidaceae alkaloids has not yet been elucidated, increasing studies based on short-read RNA-Seq technologies have led to significant discoveries of some early pathway enzymes involved in the biosynthesis of 4′-*O*-methylnorbelladine (4OMN), the last common intermediate before Amaryllidaceae alkaloid pathway branches diverge (Kilgore and Kutchan, 2016; Desgagńe-Penix, 2021). These early pathway enzymes include phenylalanine ammonia lyase (PAL, Li et al., 2018), tyrosine decarboxylase (TYDC, Hu et al., 2021), cinnamate 4-hydroxylase (C4H, Li et al., 2018), *p*-coumarate 3-hydroxylase (C3H, Hotchandani et al., 2019), norbelladine synthase (NBS, Singh et al., 2018; Tousignant et al., 2022) and norbelladine 4′-*O*-methyltransferase (N4′OMT, Kilgore et al., 2014). In addition, the oxidative coupling of 4OMN catalyzed by cytochrome P450 96T1 (CYP96T1) as well as noroxomaritidine reductase (NR) involved in the formation of oxomaritinamine have also been identified (Kilgore et al., 2016a, b). More recently, a site of Amaryllidaceae biosynthesis in daffodil tissues was successfully determined, which resulted in identifying the core biosynthetic pathways for galantamine and haemanthamine (Mehta et al., 2023).

In this study, PacBio single-molecule real-time (SMRT) sequencing of *L. aurea* was obtained by sampling different tissues and methyl jasmonate (MJ)-treated seedlings. A total of 52,338 unigenes with an average length of 1,788 bp were acquired. Among these, 49,850 unigenes were annotated in at least one public database successfully, and 380 cytochrome P450 enzyme (CYP450) genes were annotated and identified. By using homologous search, five unigenes related to oxidative-coupling cytochrome P450 were identified, and four LauCYP96Ts (including one LauCYP96T1 and three LauCYP96T1-like) genes were cloned. Moreover, functional analysis reveals that LauCYP96T1 and LauCYP96T1-like-2 is dominant for *para-para′* oxidative coupling of 4OMN, while LauCYP96T1-like-1 and LauCYP96T1-like-3 displays inverted regioselectivity, with the *para-ortho′* oxidative coupling of 4OMN. In addition, two key amino acid residues isoleucine 123 and leucine 124 in LauCYP96T1-like-1 and LauCYP96T1-like-3 were found to be responsible for the dominant changes in generated products of *para-ortho′* and *para-para′* oxidative couple. In summary, we characterized the CYP96T enzymes from *L. aurea* to elucidate the crucial reactions involved in the formation of basic scaffold that comprise the vast majority of Amaryllidaceae alkaloids.

## 2 Materials and methods

### 2.1 Plant growth and treatments

*L. aurea* bulbs were planted in the experimental field at Institute of Botany, Jiangsu Province and Chinese Academy of Sciences, Nanjing, China. Different tissues of *L. aurea* such as flower scape (sc), flower stalk (stk), petal (pe), stamen (sta), pistil (pi) and seed were sampled during the flowering time, whereas root (r), bulb (b), and leaf (L) from the same plants were collected at vigorous vegetative growth stage. In addition, one-year-old *L. aurea* seedlings grown in growth chamber were subjected into 1% dimethylsulfoxide (DMSO) solution (CK) or treated with 100 μM methyl jasmonate (MJ) for 0, 6, 12, 24 and 36 h, as described previously (Zhou et al., 2024). The samples were harvested, flash frozen in liquid nitrogen immediately, and stored at −80°C.

### 2.2 RNA extraction and detection

Total RNA was extracted using improved cetyltrimethyl ammonium bromide (CTAB) method and digested with RNase-free DNase I (Takara Bio). RNA purity was checked by electrophoresing on a 1% agarose gel. The concentration of RNA was detected using NanoDrop™ Lite spectrophotometer (Thermo Fisher Scientific) and Qubit^®^2.0 Flurometer (Life Technologies). RNA quality was also assessed with Agilent Bioanalyzer 2100 system (Agilent Technologies). Only high-quality RNA samples (A_260_/A_280_ = 1.8–2.1, A_260_/A_230_ ≥ 1.9, RIN ≥ 8.5, 28S/18S ≥1.0) were used for cDNA libraries construction.

### 2.3 PacBio cDNA library construction and SMRT sequencing

The mixed mRNA from nine different tissues as well as MJ-treated and MJ-free (CK) samples with 4 μg was reverse-transcribed into cDNA using a SMARTer PCR cDNA Synthesis Kit, and prepared to construct the PacBio SMRT-Seq library according to the protocol (OE Biotech Company). The qualified library was then sequenced on a PacBio Sequel platform, presented by Pacific Biosciences. The raw sequencing reads could be accessed from the National Center for Biotechnology Information (NCBI) database (accession number: PRJNA1106235).

### 2.4 PacBio isoform sequencing data processing

Raw sequencing data were processed using SMRTLink 6.0. The subreads data (bam format) were used to generate the circular consensus sequence (CCS, bam format). Using the default settings of isoseq3 software, the CCS reads was further used for generating the full-length (FL) and non-full-length (NFL) sequences, depending on whether the 5′ primer, 3′ primer, and poly-A tail were observed. Afterwards, the Iterative Clustering for Error Correction (ICE) technique was utilized for grouping the full-length non-chimeric (FLNC) reads. Subsequently, the high-quality polished consensus sequences are obtained with application of the Arrow algorithm. Finally, *De novo* consensus isoforms with high quality were acquired, and redundancy was removed using CD-HIT software (Fu et al., 2012) to obtain the final full-length unigenes.

### 2.5 Assessment of full-length transcriptome integrity and functional annotation

The integrity of full-length transcriptome was evaluated with Benchmarking Universal Single Copy Orthologs (BUSCO). According to OrthoDB database, BUSCO constructed single-copy gene sets of multiple evolutionary branches. The integrity and accuracy of the obtained unigenes could be more accurately determined by comparing the transcripts to the gene sets of proximate species (Simão et al., 2015). To predict putative gene function, the unigenes were mapped to the NCBI non-redundant (NR) protein sequences, SwissProt, Kyoto Encyclopedia of Genes and Genomes (KEGG), clusters of eukaryotic orthologous groups (KOG), Gene Ontology (GO), eggNOG, and Pfam databases.

### 2.6 Illumina cDNA library construction and sequencing

The RNA of twenty-seven samples from *L. aurea* different tissues and twenty-seven samples of MJ-free and MJ-treated seedlings (Zhou et al., 2024) were used for the construction of cDNA libraries with TruSeq Stranded mRNA LTSample Prep Kit (Illumina) according to the manufacturer’s instructions. Then, these libraries were sequenced on the Illumina HiSeq X Ten platform (OE Biotech Company). Raw data were filtered by trimming adaptors as well as removing low-quality reads and deposited in the NCBI BioProject database (project number: PRJNA1106651 and PRJNA1065507).

### 2.7 Gene expression analysis

By using unigenes yielded from SMRT-Seq as reference sequences, the expression level of unigenes in different tissues of *L. aurea* as well as under MJ treatments was further analyzed. By using bowtie2 software, clean data by RNA-Seq were mapped to the full-length unigene sequenced by SMRT-Seq, and reads count value of genes was obtained. All the counts were converted to Fragments Per Kilobase of transcript per Million fragments mapped (FPKM) values. Differential expression analysis of unigenes in different tissues as well as under MJ treatment was performed by hierarchical clustering on the FPKM matrix using Multi Experiment Viewer (MeV v.4.8).

### 2.8 Identification and sequence analysis of *LauCYP96T* genes

The *NpsCYP96T1* gene from *Narcissus* sp. *aff*. *Pseudonarcissus* in NCBI (accession number KT693311, Kilgore and Kutchan, 2016) was used as the query template to screen the *CYP96T* homologous genes in *L. aurea* full-length transcriptome data using the local BLASTn program with e-value of 1.0 × E^-10^ in Bioedit software. In total, five unigenes were selected for *L. aurea CYP96T* (*LauCYP96T1* and *LauCYP96T1-like*) genes, and their full-length open reading fame (ORF) sequences were analyzed. Then, these unigenes were cloned by reverse transcription PCR (RT-PCR) with specific primers (**Supplementary Table S1**; **Figure S1**). Multiple sequence alignments of *CYP96T* genes were performed using DNAMAN. In addition, the different plant *CYP96T* sequences were selected to construct the phylogenetic tree by using the MEGA software (version 5.0) with the maximum likelihood method.

### 2.9 Subcellular localization of LauCYP96T proteins

To obtain LauCYP96T-GFP fusion proteins, the ORF sequence of *LauCYP96T* (excluding the termination codon) was fused with green fluorescent protein (GFP) gene in pCAMBIA1300-GFP vector. The endoplasmic reticulum (ER) marker HDEL-mCherry was used for co-localization assay of LauCYP96T-GFP. After transforming into *Agrobacterium tumefaciens* strain GV3101, the cultured suspension cells containing each construct were then injected into leaves of 6-week-old *Nicotiana benthamiana* together. The transformed leaves were observed with a confocal laser scanning microscope (Carlzeiss 900) after growing for 48 h.

### 2.10 Transient expression of LauCYP96T genes in *N. benthamiana*

The *A. tumefaciens* harboring each construct were cultured overnight to the optical density (OD) at 600 nm of 2.0. Then, the cells were pelleted and resuspended in MMA buffer (pH 5.6) containing 10 mM 2-Morpholineethanesulfonic acid, 10 mM MgCl_2_, and 150 μM acetosyringone, to an OD_600_ of 0.4. After incubation at room temperature for 3 h, the suspensions were infiltrated into the leaves of 4-week-old *N. benthamiana*. Three days postinfiltration, the substrate 4′-*O*-methylnorbelladine (4OMN, dissolved in 0.2% DMSO) at a final concentration of 200 μM was injected. The leaves were then sampled for another day, and snap-frozen in liquid nitrogen, and freeze-dried. Samples were extracted with methanol for ten times within 24 hours at 4°C with sonicating for three times (100 Hz, 30 min each) at regular intervals. The samples centrifuged at 12,000 g for 10 minutes, and then the supernatant was filtered through a 0.22 µm sterile filter membrane and used for the subsequent analysis. Standards for 4′-*O*-methylnorbelladine, demethylnarwedine and noroxomaritidine were chemically synthesized by KaiMu Chemical Technology Co., Ltd.

### 2.11 LC–MS analysis of leaf extracts and standards

The samples were analyzed by LC-MS using an UHPLC (Agilent 1260) coupled with an Agilent 6530 quadrupole time-of-flight (qTOF) mass spectrometer with an electrospray ion source using a ZORBBAX Extend-C18 column (4.6×100 mm, 1.8 μm) (Agilent Technologies). The mobile phase consisted of solvent A (0.1% ammonium hydroxide solution in water) and solvent B (CH_3_CN) at a flow rate of 0.4 ml/min, with a column oven at 35°C. Positive ion source (ESI) was used to collect MS data. The sample was subjected to a binary gradient elution, with an initial rinse of 3% B for 1.0 min, 3-50% B from 1 min to 16 min and 50-97% B from 16 min to 17 min, 97% B from 17 min to 22 min, and 97-3% B from 22 min to 23 min, returned to initial 3% B and maintained until 26 min for column balance.

### 2.12 Molecular homolog modelling and docking

SWISSMODEL (http://swissmodel.expasy.org/) was used to display and analyze the tertiary structure of LauCYP96T1, LauYP96T1-like-1, LauCYP96T1-like-2, and LauCYP96T1-like-3. The Alphafold-predicted structure of NpsCYP96T1 (UniProt: A0A2H5AJ00; https://alphafold.ebi.ac.uk/entry/A0A2H5AJ00) was used as the template to create the model. Subsequently, Modeler and the Profiles-3D module integrated within Discovery Studio v2019 (Accelrys Inc) were employed to refine and evaluate the generated models. Discovery Studio v2019 was also used for the visualization of the 3-D models and docking analysis with 4OMN as ligands. To identify candidate residues important for regioselectivity, multiple-sequence alignments among LauCYP96T protein sequences from different plant species were performed, and the amino acid differences among LauCYP96T proteins were identified. For site-directed mutagenesis, the mutants were generated using a combination of PCR/Gibson assembly. Primer and templates are listed in **Supplementary Table S1**.

## 3 Results

### 3.1 PacBio sequencing of *L. aurea*

To generate the *L. aurea* full-length transcriptome, mixed RNA were collected and two SMRT cells were used for the PacBio Sequel platform, yielding 41.72 Gb assembled subreads, with the length ranging from 50 bp to 203,296 bp (**Supplementary Table S2**). In detail, 929,714 circular consensus sequences (CCS) were retained after filtering, and the mean length was 2,070 bp. Further, by searching for the polyA tail signal and the 5′ and 3′ cDNA primers, 735,816 full-length non-chimeric (FLNC) transcripts were identified (**Supplementary Table S3**). In addition, after FLNC cluster analysis and further error correction, 65,952 high-quality isoforms (accuracy over 99%) were obtained with N50 of 2,052 bp (**Supplementary Table S4**). Subsequently, by removing redundancy, 52,338 full-length unigenes were obtained with N50 of 2,067 bp (**Supplementary Table S4**), and used as reference sequences for further RNA-Seq analysis. Afterwards, transcriptome quality of *L. aurea* was evaluated using BUSCO. In the OrthoDB database, 85% complete BUSCOs were covered by unigenes, which confirmed the completeness of the *L. aurea* full-length transcriptome (**Supplementary Table S5**). Moreover, the coding sequences (CDs) regions were also predicted, and a total of 51,137 sequences were annotated as CDs.

### 3.2 Gene annotation and functional classification

**Figure 1:**
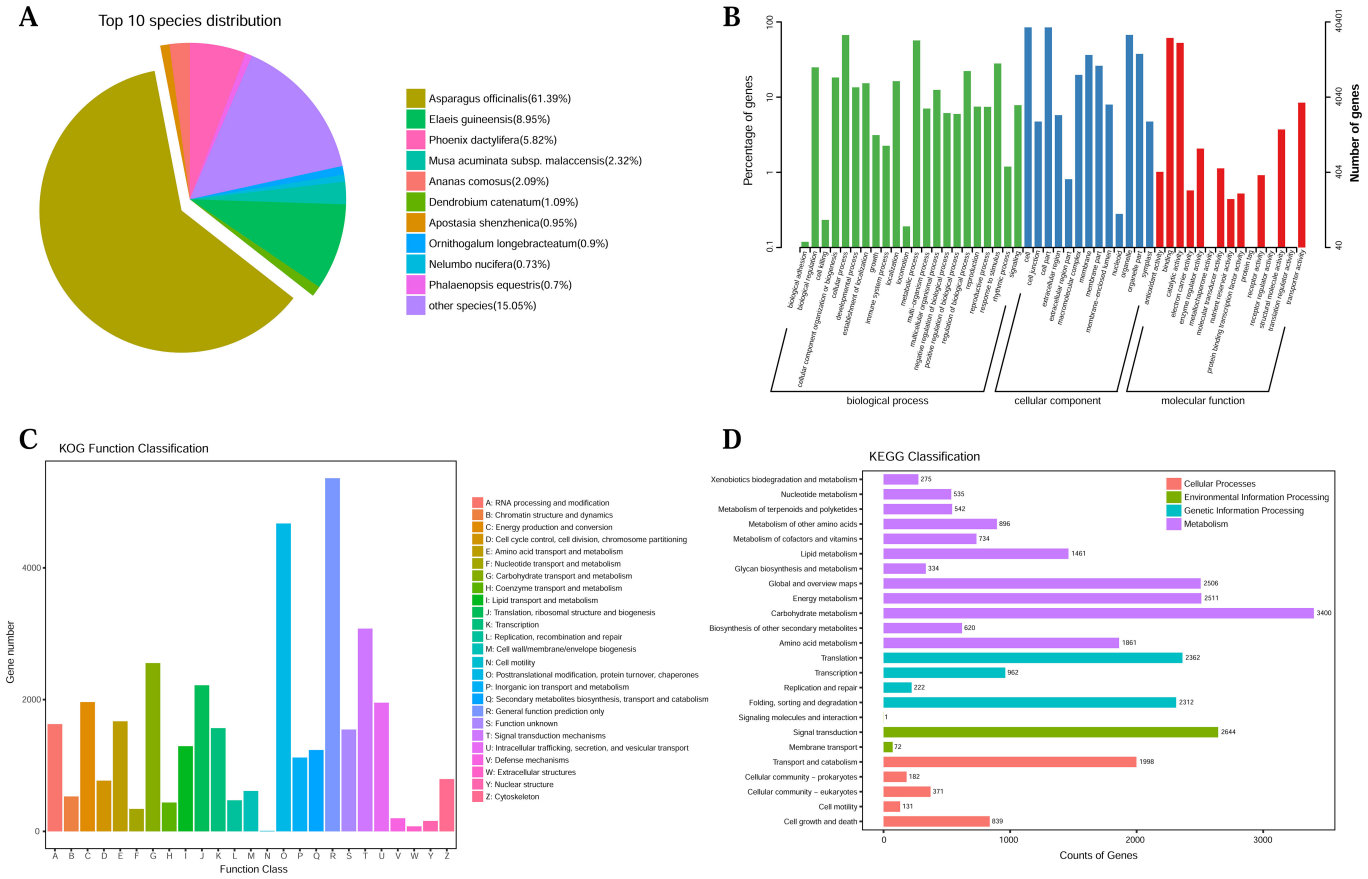
Functional annotations of full-length transcriptome for *L. aurea.* **(A)** NR homologous species distribution analysis. **(B)** GO functional classification. **(C)** KOG functional classification. **(D)** KEGG enrichment analysis.

Comprehensive functional annotation of 52,338 unigenes was performed by using BLAST search in seven public databases. It showed that 49,796 (95.14%), 42,411 (81.03%), 23,947 (45.75%), 32,418 (61.94%), 47,950 (91.62%), 40,401 (77.19%), and 43 (0.08%) unigenes were annotated based on alignment to NR, Swissprot, KEGG, KOG, eggNOG, GO, and Pfam databases, respectively (**Supplementary Table S6**). Homologous species of *L. aurea* were then predicted, and the largest number of sequences was found in *Asparagus afficinalis* (61.39%), followed by *Elaeis guineensis* (8.95%), and *Phoenix dactyliferas* (5.82%; **Figure 1A**). In the GO classification, 40,401 unigenes were clustered into three major categories and 50 subcategories (**Figure 1B**). For KOG analysis, unigenes were functionally categorized into 25 classes. Of these categories, “general function prediction only” (5,361 unigenes) was the largest enriched category (**Figure 1C**). Using the KEGG pathway database, enriched metabolic pathways were categorized. A total of 23,947 genes were mapped to the KEGG database in four categories (**Figure 1D**), where “global and overview maps” and “signal transduction” were the most abundant subcategories.

### 3.3 Expression analysis of assembled *L. aurea* transcripts in different tissues as well as under MJ treatment

To identify transcriptional responses of unigenes in different tissues and under MJ treatment, 27 cDNA libraries of nine different tissues (flower scape, flower stalk, petal, stamen, root, bulb, and leaf) from different growth stage of *L. aurea* as well as 27 cDNA libraries of *L. aurea* seedlings under MJ treatment with different time (Zhou et al., 2024) were constructed and subsequently sequenced. For 27 libraries from different tissues of *L. aurea*, a total of 191.83 Gbyte clean bases were obtained. The quality score above 30 (Q30) of each library was over 93.85%, and GC percentages was around 44.65% to 47.11% (**Supplementary Table S7**). The results were similar to that of 27 cDNA libraries of *L. aurea* seedlings under MJ treatment with different time (Zhou et al., 2024). Subsequently, the base number of clean reads were successfully mapped to unigenes of full-length transcriptome and the expression pattern of each unigene was also analyzed (**Supplementary Table S8**).

### 3.4 Identification of candidate LauCYP96Ts involved in oxidative coupling of substrate 4′-*O*-methylnorbelladine

Previous study has demonstrated that CYP96T1 is responsive for oxidatively coupling 4OMN to generate the *para*-*para*′ (*p*-*p*′) scaffold (Kilgore et al., 2016a). More recently, the CYP96T1-like cytochrome P450, *Nt*CYP96T6 and *Nt*CYP96T5, which generate the *para*-*ortho*′ (*p*-*o*′) and *ortho*-*para*′ (*o*-*p*′) intermediates were identified in *Narcissus* cv. Tête-à-Tête (Mehta et al., 2023). Based on the full-length transcriptome data, we also focused on identifying CYP450 candidates that might contribute to Amaryllidaceae alkaloids biosynthesis in *L. aurea*. By using *NpsCYP96T1* as bait to screen the *LauCYP96T* genes in full-length transcriptome data of *L. aurea*, five unique CYP450s were identified as *LauCYP96T* homolog genes (**Supplementary Figure S1**; **Supplementary Table S9**). In addition, a total of 380 CYP450 unigenes were also searched based on the annotations. Afterwards, co-expression analysis of these CYP450s was performed for hierarchical clustering. The results showed that these five *CYP96T1* homolog unigenes were clustered together (**Supplementary Figure S2**), suggesting the similar function they may have. Thus, we amplified these candidate genes with specific primers by RT-PCR (**Supplementary Table S1**; **Supplementary Figure S1**). Finally, four *LauCYP96T* genes were obtained, and named as *LauCYP96T1*, *LauCYP96T1-like-1*, *LauCYP96T1-like-2*, and *LauCYP96T1-like-3* (**Supplementary Table S9**). Afterwards, the nucleotide and protein sequence alignments from different plant species were also performed (**Supplementary Figure S3**; **Supplementary Table S10**). The results showed that these four LauCYP96Ts have high identities to previous CYP96Ts from different plant species (**Supplementary Figure S3**).

### 3.5 Subcellular localization of LauCYP96Ts

**Figure 2:**
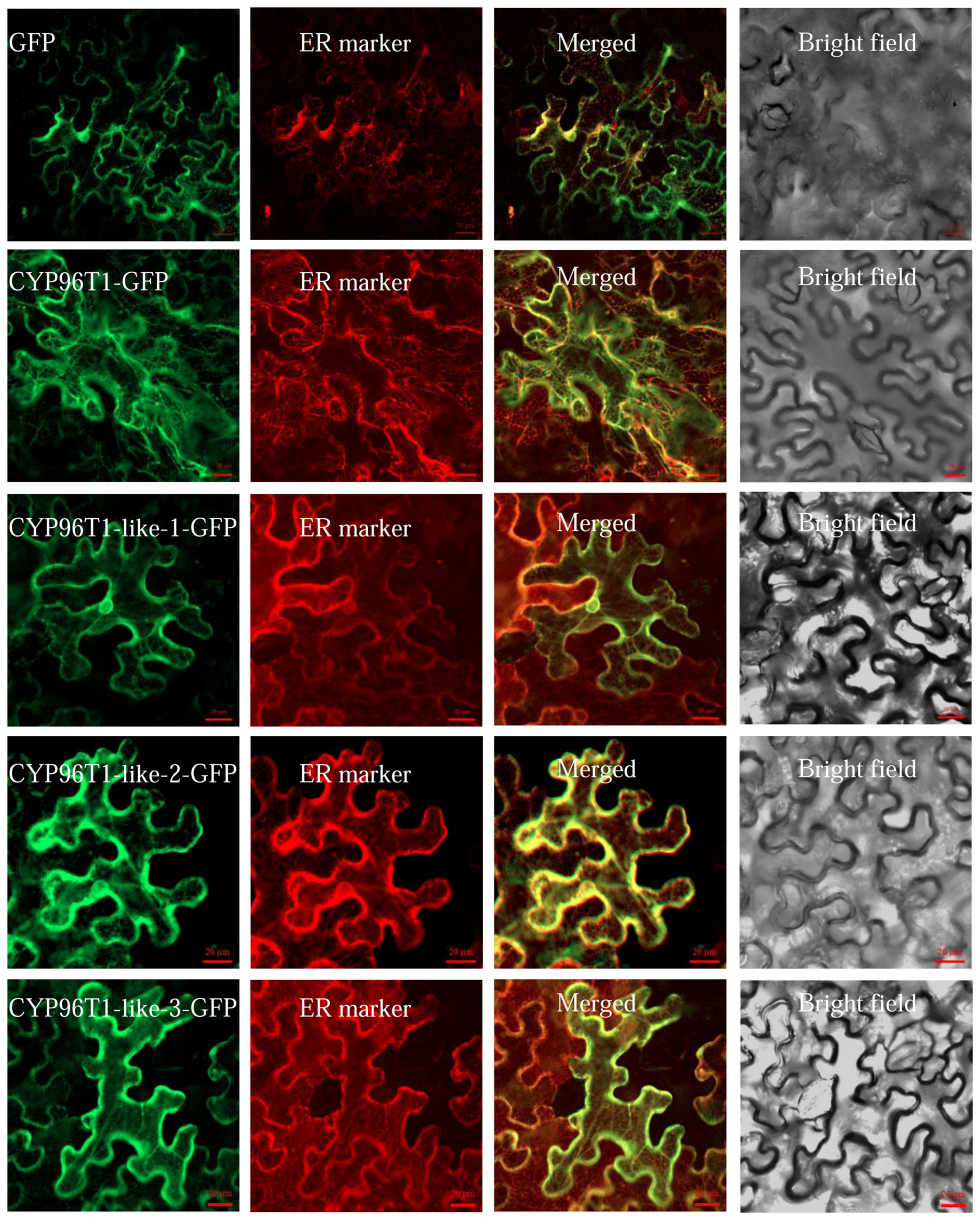
Subcellular localization of LauCYP96T proteins. LauCYP6T1, LauCYP6T1-like-1, LauCYP6T1-like-2, and LauCYP6T1-like-3 fused in-frame with green fluorescent protein (GFP), respectively, was transiently expressed in tobacco leaf cells. The 35S: HDEL-mcherry was used as a endoplasmic reticulum (ER) marker. Scale bars are 20 μm.

To investigate the subcellular localization of LauCYP96Ts, we fused the *GFP* gene with *LauCYP96T* genes individually at their C-terminals. The results showed that GFP alone were localized in the nucleus and cytosol (**Figure 2**). However, the fluorescent signal of LauCYP96T-GFP fusion proteins was extensively detected in the endoplasmic reticulum (ER), and overlapped with the red fluorescent signal of HDEL-mcherry. These data implied that LauCYP96Ts are ER-localized proteins (**Figure 2**).

### 3.6 Expression and *in vitro* enzymatic reactions of the LauCYP96T proteins in *N. benthamiana*

**Figure 3:**
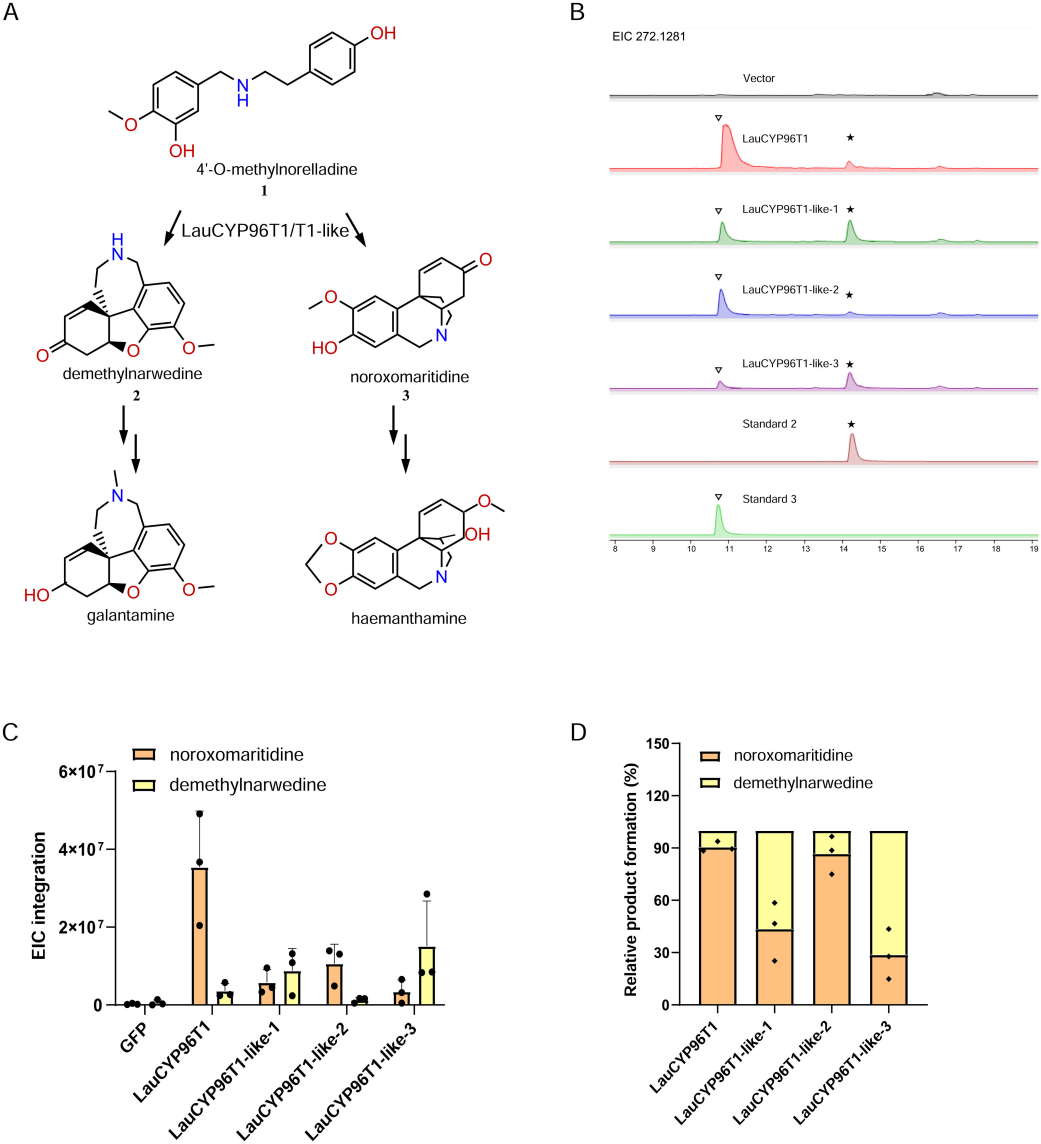
Functional characterization of LauCYP96T1 and LauCYP96T1-like proteins involved in oxidative coupling of 4′-*O*-methylnorbelladine. **(A)** The reaction scheme of LauCYP96T1 and LauCYP96T1-like for oxidative coupling of 4′-*O*-methylnorbelladine into *para*-*para*′ (*p-p*′) and *ortho-para*′ (*o-p*′) products. **(B)** Extracted ion chromatograms (EIC) for the oxidative coupling activities of LauCYP96T1 and LauCYP96T1-like proteins. Asterisk and triangle indicate the peak of demethylnarwedine and noroxomaritidine respectively. **(C)** EIC peak integrations for the [M+H]^+^ of demethylnarwedine and noroxomaritidine oxidative-coupled by LauCYP96T1 and LauCYP96T1-like proteins. **(D)** Relative product formation of demethylnarwedine and noroxomaritidine oxidative-coupled by LauCYP96T1 and LauCYP96T1-like proteins.

Subsequently, the four LauCYP96Ts were tested for their oxidative coupling activities after the *N. benthamiana* leaves co-infiltrated with the chemically synthesized 4OMN substrate (**Supplementary Figure S4**). Analysis of infiltrated leaf extracts by LC-MS confirmed that, LauCYP96T1 and LauCYP96T1-like-2 produced one major peak with the expected MS fragmentation pattern for noroxomaritidine (**Supplementary Figure S5**), the *p-p*′ product (**Figures 3B–D**), and one minor peak with the expected MS fragmentation pattern for demethylnarwedine (**Supplementary Figure S6**), the *p-o*′ product (**Figures 3B–D**), as both confirmed by the chemical standard (**Supplementary Figure S7**). However, LauCYP96T1-like-1 and LauCYP96T1-like-3, produced the major *p-o*′ intermediate demethylnarwedine, and minor *p-p*′ intermediate noroxomaritidine (**Figures 3B–D**; **Supplementary Figure S7**).

### 3.7 Product profiles of chimeras and reciprocal mutagenesis in LauCYP96Ts

**Figure 4:**
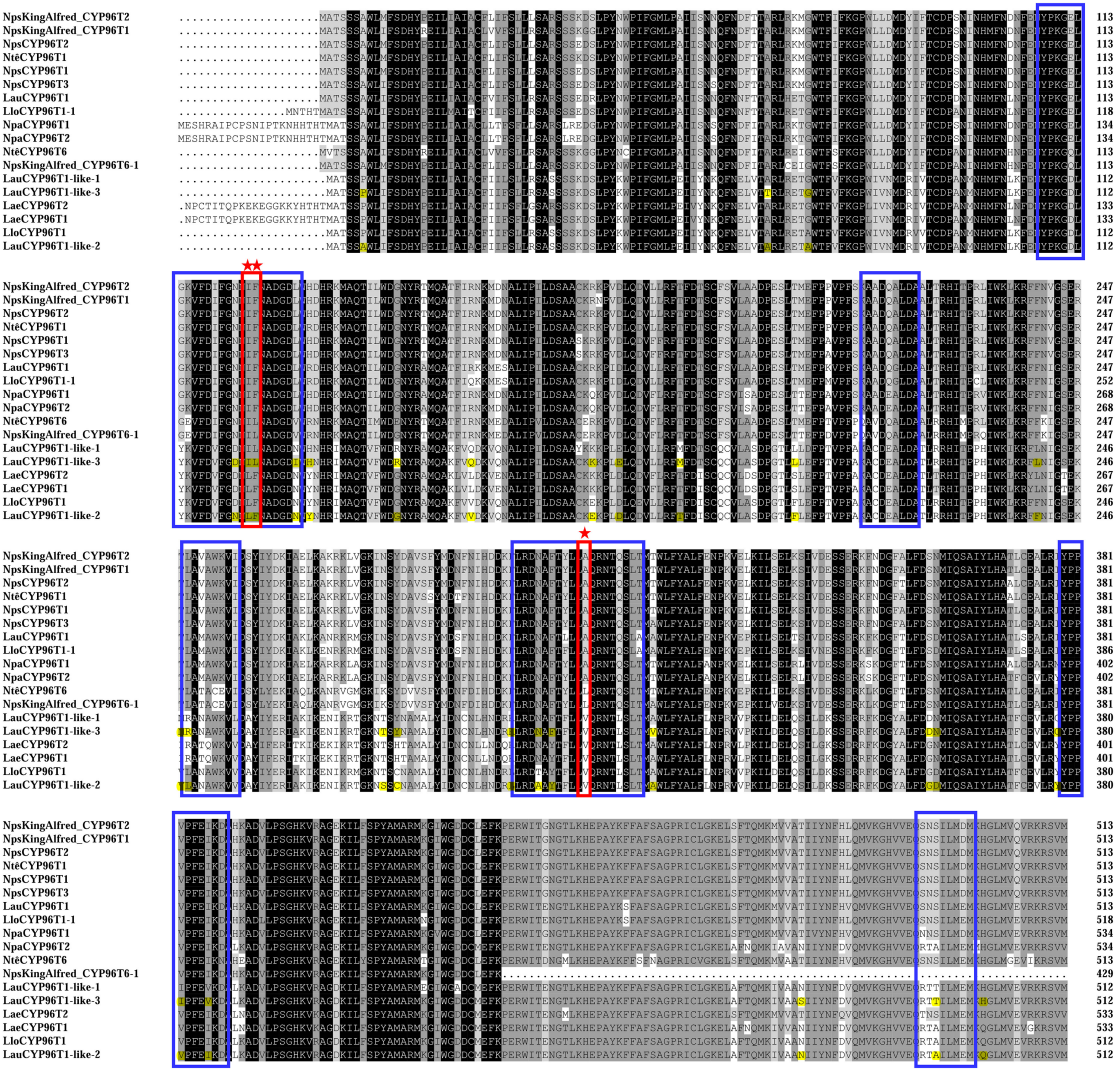
Protein sequence alignment of LauCYP96T1 and LauCYP96T1-like with homologous proteins from other species. Amino acids shaded in black are identical; those in dark and light grey are similar. Substrate recognition sites for CYP96T1 were identified, and indicated with blue frames. Possible regioselectivity-determining sites are marked with red frames and asterisks. Different amino acid resides between LauCYP96T1-like-2 and LauCYP96T1-like-3 were highlighted. The characterized CYP96T from *Narcissus* sp. *aff*. *pseudonarcissus* (NpsCYP96T), *Narcissus pseudonarcissus* var. King Alfred (NpsKingAlfred_CYP96T), *Narcissus* cv. Tête-à-Tête (NtêCYP96T), *Narcissus papyraceus* (NpaCYP96T), *Leucojum aestivum* (LaeCYP96T), and *Lycoris longituba* (LloCYP96T) were listed.

**Figure 5:**
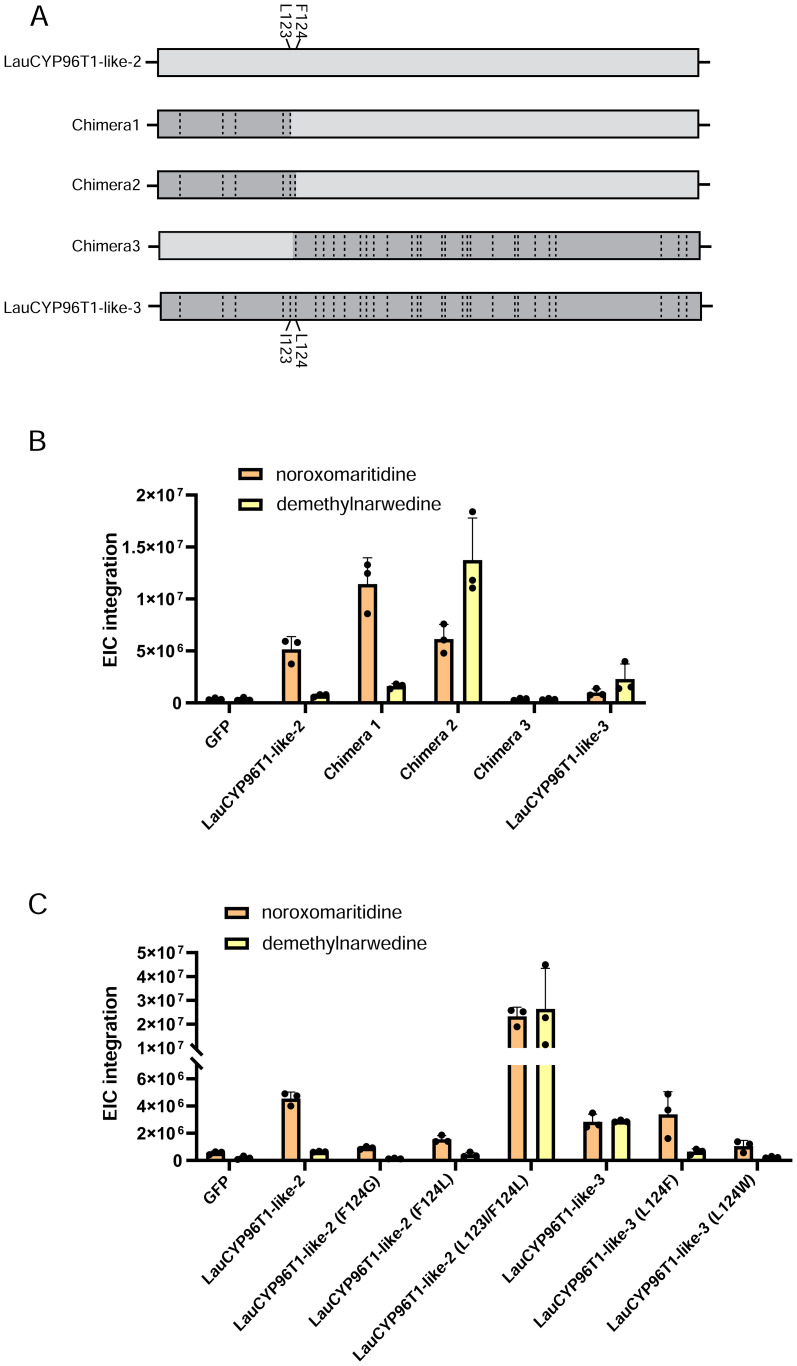
Engineered chimeric products of LauCYP96T1-like-2 and product profiles of inversion site-directed mutagenesis of residue on both LauCYP96T1-like-2 and LauCYP96T1-like-3. **(A)** Different chimeras of LauCYP96T1-like-2 and LauCYP96T1-like-3 harbor amino acid alterations (dashed lines). **(B)** Relative production of the noroxomaritidine and demethylnarwedine with infiltrated 4OMN substrate by different chimeras. **(C)** Product profiles composed of noroxomaritidine and demethylnarwedine of swapping residues 123 and 124 on LauCYP96T1-like-2 and LauCYP96T1-like-3.

Similar to previous reports (Kilgore et al., 2016a; Mehta et al., 2023), LauCYP96T1 homologous sequences (LauCYP96T1 and LauCYP96T1-like-2) produce only trace amounts of the *p-o*′ intermediate demethylnarwedine, while LauCYP96T1-like-1 and LauCYP96T1-like-3 displays inverted regioselectivity, with the *p-o*′ intermediate demethylnarwedine as the predominant product (**Figure 3**). Then, we sought to identify the key amino acid residues involved in the catalytic enzyme activity of identified LauCYP96T. In addition, protein sequence alignments showed that CYP96Ts from different species were highly similar to each other, exhibiting above 70% identity at the amino acid level (**Figure 4**). Thus, we looked for amino-acid differences among sequences of orthologous proteins that resided within the substrate recognition sites (SRSs)-motifs that are characterized previously (Mehta et al., 2023). By comparing the amino acid sequences of CYP96Ts, a differential residue (phenylalanine 125 in LauCYP96T1; phenylalanine 124 in LauCYP96T1-like-2; leucine 124 in LauCYP96T1-like-1 and LauCYP96T1-like-3) was found. Considering the higher homologue and inverted regioselectivity between LauCYP96T1-like-2 and LauCYP96T1-like-3, a set of chimeras (chimera 1, chimera 2 and chimera 3) were constructed to determine whether phenylalanine 124 involved in affecting their products formation (**Figure 5A**). All chimeric enzymes consist of LauCYP6T1-like-2 with sequence substitutions from LauCYP6T1-like-3 (**Figure 5A**; **Supplementary Table S11**). Compared to LauCYP6T1-like-2, although the major *p-p*′ intermediate noroxomaritidine increased, no significant change of *p-o*′ intermediate demethylnarwedine was observed in chimera 1. Conversely, the *p-o*′ intermediate demethylnarwedine significantly increased in chimera 2 comparing to LauCYP6T1-like-2 (**Figure 5B**). Above results suggested that phenylalanine 124 in LauCYP96T1-like-2 might be important for the production of different *p-p*′ and *p-o*′ intermediate. Surprisingly, chimera 3 showed no catalytic enzyme activity towards to both the production of *p-o*′ intermediate demethylnarwedine and *p-p*′ intermediate noroxomaritidine (**Figure 5B**).

We next assessed the impact of swapped amino acid residue 124 in LauCYP96T1-like-2 and LauCYP96T1-like-3 on catalytic regioselectivity, and tested these mutants in the transient expression system in *N. benthamiana*. As expected, when the single leucine 124 (L124) from LauCYP96T1-like-3 was exchanged to phenylalanine (F) or tryptophan (W), the production of *p-o*′ intermediate demethylnarwedine was dramatically decreased (**Figure 5C**). When the single F124 on LauCYP96T1-like-2 mutated to the glycine 124 (G124) or corresponding L124 from LauCYP96T1-like-3, the *p-o*′ oxidative coupling ability of LauCYP96T1-like-2 did not improved, and it even impart LauCYP96T1-like-2 with the decreased ability to catalyze the *p-p*′ oxidative coupling (**Figure 5C**). However, a significant increase of both *p-o*′ intermediate demethylnarwedine and *p-p*′ intermediate noroxomaritidine was observed, when L123 and F124 in LauCYP96T1-like-2 were changed to isoleucine 123 (I123) and L124 accordingly (**Figure 5C**).

### 3.8 Structural homology modeling reveals the involvement of amino acid in LauCYP96T for divergent regioselectivities of oxidative coupling

**Figure 6:**
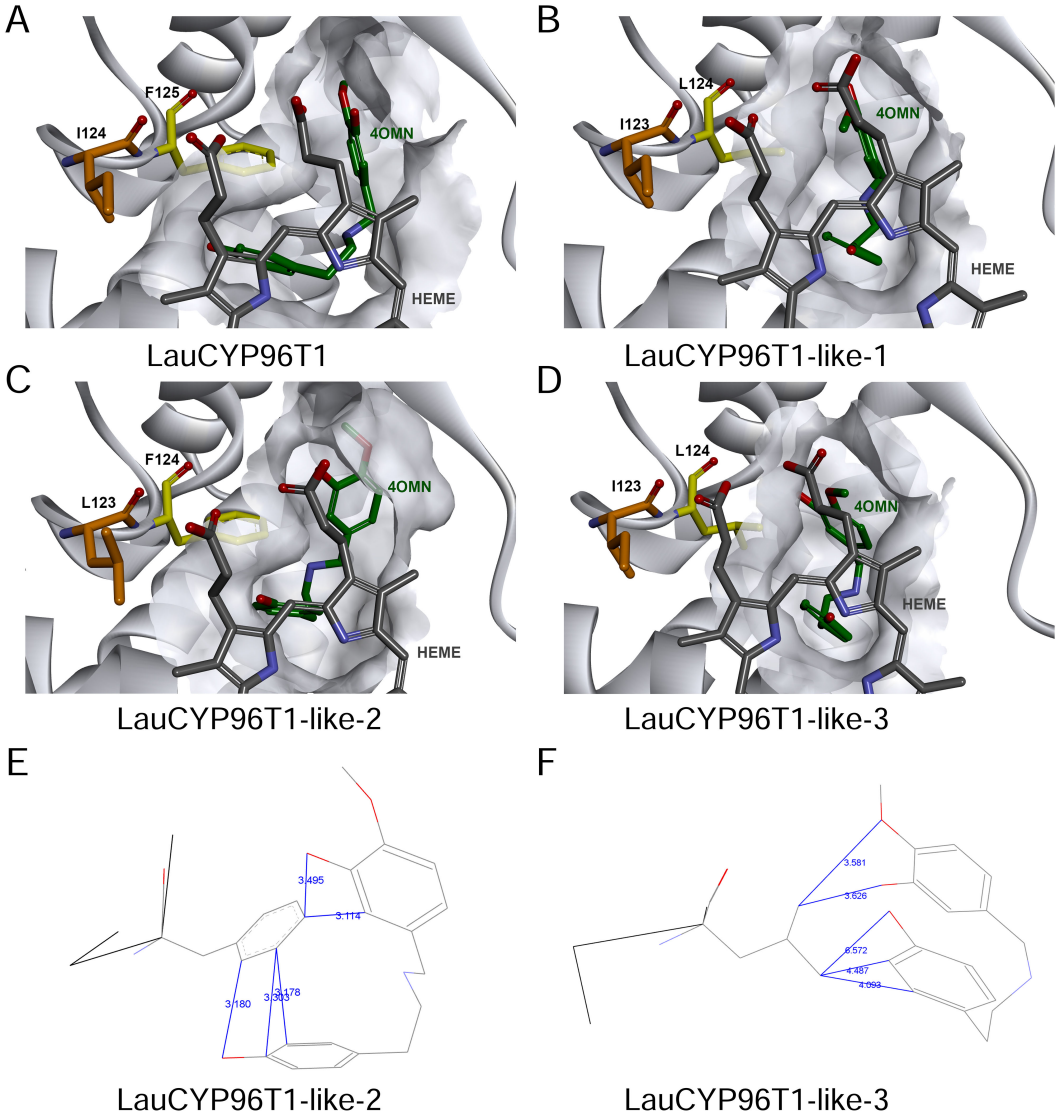
Molecular modeling and substrate docking studies support the distinct catalysis of LauCYP96T1-like-2 in comparison to LauCYP96T1-like-3. Active center architectures of LauCYP96T1 **(A)**, LauCYP96T1-like-1 **(B)**, LauCYP96T1-like-2 **(C)**, and LauCYP96T1-like-3 **(D)** with docked substrate 4′-*O*-methylnorbelladine. The common substrate pocket is shown in light grey. Substrate 4′-*O*-methylnorbelladine and cofactor heme was highlighted as green and grey, respectively. In addition, phenylalanine 125 in LauCYP96T1, phenylalanine 124 in LauCYP96T1-like-2, leucine 124 in LauCYP96T1-like-1 and LauCYP96T1-like-3 was highlighted as yellow; isoleucine 124 in LauCYP96T1, leucine 123 in LauCYP96T1-like-2, isoleucine 123 in LauCYP96T1-like-1 and LauCYP96T1-like-3 was highlighted as orange; Possible effects of phenylalanine 124 in LauCYP96T1-like-2 **(E)** and leucine 124 in LauCYP96T1-like-3 **(F)** on the fold pattern of substrate 4′-*O*-methylnorbelladine were also shown. The distances between atoms of residues and substrate are indicated in Å.

Subsequently, homology modelling and substrate docking of the LauCYP96T1, LauCYP96T1-like-1, LauCYP96T1-like-2, and LauCYP96T1-like-3 using NpCYP96T1 as a template (UniProt identifier: A0A2H5AJ00) were employed to see where reciprocally changed amino acids are situated in the enzyme gain insights into the close interactions of the substrate with the enzyme active center (**Supplementary Figure S8**). As shown in **Figures 6**, amino acid residue L124 in LauCYP96T1-like-1 and LauCYP96T1-like-3 (corresponding to F125 in LauCYP96T1 and F124 in LauCYP96T1-like-2) locates close to the active center and the substrate, which could explain the findings in our mutagenesis studies. In addition, amino acid residue I123 in LauCYP96T1-like-1 and LauCYP96T1-like-3 (corresponding to I124 in LauCYP96T1 and L123 in LauCYP96T1-like-2) locates close to the cofactor heme, which might have effects on the heme-binding sites.

## 4 Discussion

Due to a lack of genetic references and suitable model of uncovering developmental correlate to metabolite biosynthesis in Amaryllidaceae family, the molecular mechanisms for underlying the complete pathway of Amaryllidaceae alkaloids biosynthesis have not been fully understood. Recently, a few researchers have used short-read RNA-seq technologies to investigate the key enzyme genes involved in this pathway in Amaryllidoieae family (Kilgore et al., 2014, Kilgore et al., 2016a, b; Wang et al., 2017; Li et al., 2021). However, fragments generated by short-read RNA-seq often result in erroneous transcript structure and misleading expression levels of the different transcription variants (Steijger et al., 2013; Bernard et al., 2014). Thus, the full-length sequencing technology may generate longer reads than short-read RNA-seq methods, which could improve the accuracy of the transcript data (Rhoads and Au, 2015). More recently, a complete set of biosynthetic genes for the production of Amaryllidaceae has been revealed from daffodil long-read PacBio IsoSeq database (Mehta et al., 2023).

To further understand the potential biosynthesis pathway of Amaryllidaceae alkaloids in *L. aurea*, we also performed full-length transcriptome sequencing by sampling different tissues as well as MJ-treated seedlings of *L. aurea*. In total, 735,816 FLNC transcripts were obtained from SMRT sequencing, and 52,338 non-redundant unigenes were generated. Our study presents high-quality transcriptome data for *L. aurea*, which will also provide valuable information for further studies on the molecular mechanisms of different processes in *L. aurea.* Thus, the expression pattern of each unigene was analyzed. With regards to galantamine, CYP96T1 and CYP96T6 have been shown to catalyze the *para-ortho*′ coupling of 4OMN to produce the galantamine precursor demethylnarwedine (Kilgore et al., 2016b; Mehta et al., 2023). In addition, considering the high identity between CYP96T1 and CYP96T6 (Mehta et al., 2023), we thus blastn-searched the homologous genes of *NpsCYP96T1* in *L. aurea*, and five candidate genes were identified (including four unigenes with complete ORFs). In total, four *LauCYP96T* genes including *LauCYP96T1*, *LauCYP96T1-like-1, LauCYP96T1-like-2*, and *LauCYP96T1-like-3* were obtained by RT-PCR, and subsequently functionally verified by *in vitro* enzymatic reactions in *N. benthamiana*. Similar to NpsCYP96T1 and NtêCYP96T1, LauCYP96T1 and LauCYP96T1-like-2 function mainly in the production of *p-p*′ intermediate noroxomaritidine, while LauCYP96T1-like-1 and LauCYP96T1-like-3 have significant abilities for improving *p-o*′ intermediate demethylnarwedine production (**Figure 3**).

In *Narcissus* cv. Tête-à-Tête, NtêCYP96T6 has been demonstrated to be responsible for the *p-o*′ intermediate demethylnarwedine production (Mehta et al., 2023). For identification of the regioselectivity-determining residues in LauCYP96T-like homologs, amino acid sequence alignments among CYP96T proteins from different plant species were performed (Mehta et al., 2023). We noticed that one amino acid (take F124 in LauCYP96T1-like-2 and L124 in LauCYP96T1-like-3 for example) is identity among LauCYP96T1-like-1, LauCYP96T1-like-3, NtêCYP96T6, and NpsKingAlfred_CYP96T6-1, while different within other CYP96Ts (**Figure 4**). Accordingly, when chimeras and reciprocal mutagenesis are referring to the changes of amino acid residue 124 on LauCYP96T1-like-2 and LauCYP96T1-like-3, the regioselectivity activity varied. However, the neighbor amino acid residue 123 is also connected with regioselectivity for oxidative coupling of 4OMN, which was proven by the activity of chimera 3 and LauCYP96T1-like-2 (L123I/F124L). Moreover, it revealed that the alanine 308 (A308) site present within the oxygen-binding motif of NtêCYP96T1, is a key regioselectivity-determining residue on *Nt*CYP96T1 due to its close spatial proximity to the predicted binding site for its substrate 4OMN (Mehta et al., 2023). We also noticed the conserved A308 site in LauCYP96T1 (**Figure 4**). However, the corresponding A307 in other LauCYP96T1-like proteins has been changed to valine307 (V307), suggesting a complex oxidative coupling in different LauCYP96T variants might be existed, and could be elucidated with the crystal structures. Alternatively, the site-swapping mutagenesis based on the A308 site and phenylalanine 125 in LauCYP96T1 should be further investigated. Moreover, NtêCYP96T5, another CYP96T1-like cytochrome P450, together with the short-chain dehydrogenase/reductase NtêSDR1, catalyzing the *ortho*-*para*′ oxidative coupling of 4OMN to produce intermediate norpluviine, has also been characterized (Mehta et al., 2023). Thus, how CYP96T1-homologs have evolved to catalyze alternate modes of oxidative coupling from a common substrate should be further investigated. On the other hand, gene-coexpression analysis showed that five *CYP96T* homolog genes were clustered together among all the CYP450s (**Supplementary Figure S2**; **Supplementary Table S8**), suggesting our RNA-seq expression profiling might partly provide possibility to identifying candidate pathway genes of Amaryllidaceae alkaloids biosynthesis by co-expression analysis. More recently, biosensor and machine learning-aided engineering of norbelladine 4′-*O*-methyltransferase has been performed (d’Oelsnitz et al., 2024). Considering the core biosynthetic pathways for haemanthamine and galantamine have been revealed (Mehta et al., 2023), the engineering of enzymes in the AA biosynthesis pathway for industry-scale biomanufacturing will be accelerated.

## Data Availability

The datasets presented in this study can be found in online repositories. The names of the repository/repositories and accession number(s) can be found in the article/**Supplementary Material**.

## References

[B1] Berkov S. Georgieva L. Kondakova V. Atanassov A. Viladomat F. Bastida J.. (2009). Plant sources of galanthamine: phytochemical and biotechnological aspects. Biotechnol. Biotechnol. Equip. 23, 1170–1176. doi: 10.1080/13102818.2009.1081kil7633

[B2] Bernard E. Jacob L. Mairal J. Vert J. (2014). Efficient RNA isoform identification and quantification from RNA-seq data with network flows. Bioinformatics 30, 2447–2455. doi: 10.1093/bioinformatics/btu317 24813214 PMC4147886

[B3] Cheng B. Wang Q. An Y. Chen F. (2024). Recent advances in the total synthesis of galantamine, a natural medicine for Alzheimer's disease. Nat. Prod. Rep. 41, 1060–1090. doi: 10.1039/D4NP00001C 38450550

[B4] Chiocchio I. Mandrone M. Tomasi P. Marincich L. Poli F. (2021). Plant secondary metabolites: an opportunity for circular economy. Molecules. 26, 495. doi: 10.3390/molecules26020495 33477709 PMC7831927

[B5] d’Oelsnitz S. Diaz D. J. Kim W. Acosta D. J. Dangerfield T. L. Schechter M. W.. (2024). Biosensor and machine learning-aided engineering of an amaryllidaceae enzyme. Nat. Commun. 15, 2084. doi: 10.1038/s41467-024-46356-y 38453941 PMC10920890

[B6] Desgagńe-Penix I. (2021). Biosynthesis of alkaloids in Amaryllidaceae plants: a review. Phytochem. Rev. 20, 409–431. doi: 10.1007/s11101-020-09678-5

[B7] Fu L. Niu B. Zhu Z. Wu S. Li W. (2012). CD-hit: accelerated for clustering the next-generation sequencing data. Bioinformatics 28, 3150–3152. doi: 10.1093/bioinformatics/bts565 23060610 PMC3516142

[B8] Hotchandani T. de Villers J. Desgagné-Penix I. (2019). Developmental regulation of the expression of Amaryllidaceae alkaloid biosynthetic genes in *Narcissus papyraceus* . Genes 10, 594. doi: 10.3390/genes10080594 31394782 PMC6723416

[B9] Hu J. Li W. Liu Z. Zhang G. Luo Y. (2021). Molecular cloning and functional characterization of tyrosine decarboxylases from galanthamine-producing *Lycoris radiata* . Acta Physiol. Plant 43, 84. doi: 10.1007/s11738-021-03258-6

[B10] Jin Z. Yao G. (2019). Amaryllidaceae and sceletium alkaloids. Nat. Prod. Rep. 36, 1462–1488. doi: 10.1039/C8NP00055G 30707215

[B11] Kilgore M. B. Augustin M. M. May G. D. Crow J. A. Kutchan T. M. (2016a). CYP96T1 of Narcissus sp. aff. *pseudonarcissus* catalyzes formation of the *para-para*′ C–C phenol couple in the Amaryllidaceae alkaloids. Front. Plant Sci. 7. doi: 10.3389/fpls.2016.00225 PMC4766306 26941773

[B12] Kilgore M. B. Augustin M. M. Starks C. M. O’Neil-Johnson M. May G. D. Crow J. A.. (2014). Cloning and characterization of a norbelladine 4′-O-methyltransferase involved in the biosynthesis of the Alzheimer’s drug galanthamine in Narcissus sp. aff. *Pseudonarcissus* . PLoS One 9, e103223. doi: 10.1371/journal.pone.0103223 25061748 PMC4111509

[B13] Kilgore M. B. Holland C. Jez J. M. Kutchan T. M. (2016b). Identification of a noroxomaritidine reductase with Amaryllidaceae alkaloid biosynthesis related activities. J. Biol. Chem. 291, 16740–16752. doi: 10.1074/jbc.M116.717827 27252378 PMC4974387

[B14] Kilgore M. B. Kutchan T. M. (2016). The Amaryllidaceae alkaloids: biosynthesis and methods for enzyme discovery. Phytochem. Rev. 15, 317–337. doi: 10.1007/s11101-015-9451-z 27340382 PMC4914137

[B15] Li Q. Xu J. Zheng Y. Zhang Y. Cai Y. (2021). Transcriptomic and metabolomic analyses reveals that exogenous methyl jasmonate regulates galanthamine biosynthesis in *Lycoris longituba* seedlings. Front. Plant Sci. 12. doi: 10.3389/fpls.2021.713795 PMC8514708 34659286

[B16] Li W. Yang Y. Qiao C. Zhang G. Luo Y. (2018). Functional characterization of phenylalanine ammonia-lyase- and cinnamate 4-hydroxylase-encoding genes from *Lycoris radiata*, a galanthamine-producing plant. Int. J. Biol. Macromol 117, 1264–1279. doi: 10.1016/j.ijbiomac.2018.06.046 29894786

[B17] Liu X. Zhang P. Zhao Q. Huang A. C. (2023). Making small molecules in plants: A chassis for synthetic biology-based production of plant natural products. J. Integr. Plant Biol. 65, 417–443. doi: 10.1111/jipb.13330 35852486

[B18] Liu Y. Zhao X. Gan F. Chen X. Deng K. Crowe S. A.. (2024). Complete biosynthesis of QS-21 in engineered yeast. Nature. 629, 937–944. doi: 10.1038/s41586-024-07345-9 PMC11111400 38720067

[B19] Marco-Contelles J. do Carmo Carreiras M. Rodríguez C. Villarroya M. García A. (2006). Synthesis and pharmacology of galantamine. Chem. Rev. 106, 116–133. doi: 10.1002/chin.200618253 16402773

[B20] Mehta N. Meng Y. Zare R. Kamenetsky-Goldstein R. Sattely E. (2023). A developmental gradient reveals biosynthetic pathways to eukaryotic toxins in monocot geophytes. BioRxiv. doi: 10.1101/2023.05.12.540595 PMC11893076 39276773

[B21] Mucke H. A. (2015). The case of galantamine: repurposing and late blooming of a cholinergic drug. Future Sci. OA 1, FSO73. doi: 10.4155/fso.15.73 28031923 PMC5137937

[B22] Paddon C. J. Westfall P. J. Pitera D. J. Benjamin K. Fisher K. McPhee D.. (2013). High-level semi-synthetic production of the potent antimalarial artemisinin. Nature 496, 528–532. doi: 10.1038/nature12051 23575629

[B23] Rhoads A. Au K. F. (2015). PacBio sequencing and its applications. Genomics Proteomics Bioinf. 13, 278–289. doi: 10.1016/j.gpb.2015.08.002 PMC4678779 26542840

[B24] Simão F. A. Waterhouse R. M. Ioannidis P. (2015). BUSCO: assessing genome assembly and annotation completeness with single-copy orthologs. Bioinformatics. 31, 3210–3212. doi: 10.1093/bioinformatics/btv351 26059717

[B25] Singh A. Massicotte M. A. Garand A. Tousignant L. Ouellette V. Bérubé G.. (2018). Cloning and characterization of norbelladine synthase catalyzing the first committed reaction in Amaryllidaceae alkaloid biosynthesis. BMC Plant Biol. 18, 338. doi: 10.1186/s12870-018-1570-4 30526483 PMC6286614

[B26] Srinivasan P. Smolke C. D. (2020). Biosynthesis of medicinal tropane alkaloids in yeast. Nature 585, 614–619. doi: 10.1038/s41586-020-2650-9 32879484 PMC7529995

[B27] Steijger T. Abril J. F. Engstrom P. G. Kokocinski F. Hubbard T. J. Guigo R.. (2013). Assessment of transcript reconstruction methods for RNA-seq. Nat. Methods 10, 1177–1184. doi: 10.1038/nmeth.2714 24185837 PMC3851240

[B28] Tousignant L. Diaz-Garza A. M. Majhi B. B. Gélinas S. E. Singh A. Desgagne-Penix I. (2022). Transcriptome analysis of Leucojum aestivum and identification of genes involved in norbelladine biosynthesis. Planta 255, 30. doi: 10.1007/s00425-021-03741-x 34981205

[B29] Wang S. Li Y. He L. Yang J. Fernie A. R. Luo J. (2022). Natural variance at the interface of plant primary and specialized metabolism. Curr. Opin. Plant Biol. 67, 102201. doi: 10.1016/j.pbi.2022.102201 35349968

[B30] Wang R. Xu S. Wang N. Xia B. Jiang Y. Wang R. (2017). Transcriptome analysis of secondary metabolism pathway, transcription factors, and transporters in response to methyl jasmonate in Lycoris aurea. Front. Plant Sci. 7. doi: 10.3389/fpls.2016.01971 PMC5217099 28111578

[B31] Zhang J. Hansen L. G. Gudich O. Viehrig K. Lassen L. M. M. Schrübbers L.. (2022). A microbial supply chain for production of the anticancer drug vinblastine. Nature 609, 341–347. doi: 10.1038/s41586-022-05157-3 36045295 PMC9452304

[B32] Zhao Y. Liu G. Yang F. Liang Y. Gao Q. Xiang C.. (2023). Multilayered regulation of secondary metabolism in medicinal plants. Mol. Hortic. 3, 11. doi: 10.1186/s43897-023-00059-y 37789448 PMC10514987

[B33] Zhou Z. Wu M. Sun B. Li J. Li J. Liu Z.. (2024). Identification of transcription factor genes responsive to MeJA and characterization of a LaMYC2 transcription factor positively regulates lycorine biosynthesis in *Lycoris aurea* . J. Plant Physiol. 296, 154218. doi: 10.1016/j.jplph.2024.154218 38490054

