# Supplementary material for: Functional characterization of CYP96T1-like cytochrome P450 from Lycoris aurea catalyzing para-para′ and para-ortho′ oxidative coupling in Amaryllidaceae alkaloids biosynthesis
Source: Front Plant Sci. 2024 Oct 2;15:1438102. doi: 10.3389/fpls.2024.1438102 (PMC11479993; doi:10.3389/fpls.2024.1438102)
Supplement: Supplementary file 1 [file DataSheet1.pdf]

Supplementary Material Legends

Supplementary Table S1 List and information of primers used in this study.

Mutant name	Forward primer (5'–3')	Reverse primer (5'–3')	Template used
CYP96T1-clone1	CACACAATGGCCACTTCTTC	AATCTAATCTCACATGACTG	cDNA of *L. aurea*
CYP96T1-clone2	CACACAATGGCCACTTCTTC	TTACATGACTGATCTCTTTC	cDNA of *L. aurea*
CYP96T1-clone3	CACACAATGGCCACTTCTTC	TTACATGACTGATCTCTTTC	cDNA of *L. aurea*
CYP96T1-clone4	CACACAATGGCCACTTCTTC	AATCTAATCTCACATGACTG	cDNA of *L. aurea*
pCAMBIA1300-CYP96T1	CTCGATACACCAAATCGACTCTAGAATGGCCACTTCTTCT	GCCCTTGCTCACCATGGTACCCATGACTGATCTCTT	LauCYP96T1
pCAMBIA1300-CYP96T1-like-1	CTCGATACACCAAATCGACTCTAGAATGGCCACTTCTTCT	GCCCTTGCTCACCATGGTACCCATGACTGATCTCTT	LauCYP96T1-like-1
pCAMBIA1300-CYP96T1-like-2	CTCGATACACCAAATCGACTCTAGAATGGCCACTTCTTCT	GCCCTTGCTCACCATGGTACCCATGACTGATCTCTT	LauCYP96T1-like-2
pCAMBIA1300-CYP96T1-like-3	CTCGATACACCAAATCGACTCTAGAATGGCCACTTCTTCT	GCCCTTGCTCACCATGGTACCCATGACTGATCTCTT	LauCYP96T1-like-3
LauCYP96T1-like-2(F124L)	CTCGATACACCAAATCGACTCTAGAATGGCCACTTCTTCT	TATCTCCATCGGCGTTGAGGAGATTATTGCCGAATA	LauCYP96T1-like-2
	TCCTCAACGCCGATGGAGATAATTGGTACAACCATC	GCCCTTGCTCACCATGGTACCCATGACTGATCTCTT	LauCYP96T1-like-2
LauCYP96T1-like-2(F124G)	CTCGATACACCAAATCGACTCTAGAATGGCCACTTCTTCT	TCCATCGGCGTTGTTGAGATTAT	LauCYP96T1-like-2
	ATAATCTCGGCAACGCCGATGGA	GCCCTTGCTCACCATGGTACCCATGACTGATCTCTT	LauCYP96T1-like-2
LauCYP96T1-like-3(L124F)	CTCGATACACCAAATCGACTCTAGAATGGCCACTTCTTCT	TATCTCCATCGGCGTTGAAGATATTATCGCCGAATA	LauCYP96T1-like-3
	TCTTCAACGCCGATGGAGATATTTGGCATAACCATC	GCCCTTGCTCACCATGGTACCCATGACTGATCTCTT	LauCYP96T1-like-3
LauCYP96T1-like-3(L124W)	CTCGATACACCAAATCGACTCTAGAATGGCCACTTCTTCT	TCCATCGGCGTTACCGATATTAT	LauCYP96T1-like-3
	ATAATATCTGGAACGCCGATGGA	GCCCTTGCTCACCATGGTACCCATGACTGATCTCTT	LauCYP96T1-like-3
LauCYP96T1-like-2(LF123IL)	CTCGATACACCAAATCGACTCTAGAATGGCCACTTCTTCT	CATCGGCGTTGAGGATATTATTGCCGAATACATCGA	LauCYP96T1-like-2
	ATAATATCCTCAACGCCGATGGAGATAATTGGTAC	GCCCTTGCTCACCATGGTACCCATGACTGATCTCTT	LauCYP96T1-like-2
CHIMERA-1	CTCGATACACCAAATCGACTCTAGAATGGCCACTTCTTCT	TATCTCCATCGGCGTTGAAGATATTATCGCCGAATA	LauCYP96T1-like-3
	TCTTCAACGCCGATGGAGATAATTGGTACAACCATC	GCCCTTGCTCACCATGGTACCCATGACTGATCTCTT	LauCYP96T1-like-2
CHIMERA-2	CTCGATACACCAAATCGACTCTAGAATGGCCACTTCTTCT	TATCTCCATCGGCGTTGAGGATATTATCGCCGAATA	LauCYP96T1-like-3
	TCCTCAACGCCGATGGAGATAATTGGTACAACCATC	GCCCTTGCTCACCATGGTACCCATGACTGATCTCTT	LauCYP96T1-like-2
CHIMERA-3	CTCGATACACCAAATCGACTCTAGAATGGCCACTTCTTCT	TATCTCCATCGGCGTTGAGGAGATTATTGCCGAATA	LauCYP96T1-like-2
	TCCTCAACGCCGATGGAGATATTTGGCATAACCATC	GCCCTTGCTCACCATGGTACCCATGACTGATCTCTT	LauCYP96T1-like-3

Supplementary Table S2 Statistical analysis of raw data of full-length transcriptome sequencing of *L. aurea*.

Sample	Total reads	Total base (bp)	Minimum length	Maximum length	Mean length
SMRT_cell1	14686401	23202380425	50	118745	1579.85
SMRT_cell2	10947215	18519894075	50	203296	1691.74

Supplementary Table S3 Summary of Circular Consensus Sequence (CCS) reads and classification of transcripts.

Term	Number
CCS reads	929714
Number of CCS bases	1924590181
CCS reads length (mean)	2070
Reads with 5 and 3 Primers	764437
Non-Concatamer Reads with 5 and 3 Primers	737499
Non-Concatamer Reads with 5' and 3' Primers and Poly-A Tail	735816

Supplementary Table S4 Statistical analysis of isoform and unigene.

Sample	Total number	Total base (bp)	Minimum length	Maximum length	Mean length	N50
Isoform	65952	118584283	65	9500	1798.04	2052
Unigene	52338	93561732	65	9500	1787.64	2067

Supplementary Table S5 Summary of BUSCO assessment.

Class	Number	Percentage
Complete BUSCOs	262	86.4%
Complete and single-copy BUSCOs	94	31.0%
Complete and duplicated BUSCOs	168	55.4%
Fragmented BUSCOs	7	2.3%
Missing BUSCOs	34	11.3%
Total BUSCO groups searched	303	100%

Supplementary Table S6 Annotation of unigenes against seven public databases.

Annotation database	Number	Percentage
NR	49796	95.14 %
Swissprot	42411	81.03 %
KEGG	23947	45.75 %
KOG	32418	61.94 %
eggNOG	47950	91.62 %
GO	40401	77.19 %
Pfam	43	0.08 %
Annotated in at least one database	49850	95.25 %

Supplementary Table S7 Summary of assembly and analysis of different tissues of *L. aurea* transcriptome sequencing.

Sample	Replicate	Raw reads	Clean reads	Clean bases	Q20 (%)	Q30 (%)	GC (%)
Root	r1	49,033,938	47,404,402	7,110,660,300	97.55%	94.08%	45.49%
	r2	50,203,974	48,535,550	7,280,332,500	97.61%	94.20%	45.53%
	r3	50,097,782	48,440,162	7,266,024,300	97.51%	94.20%	46.06%
Bulb	b1	46,354,294	44,888,062	6,733,209,300	97.76%	94.51%	44.65%
	b2	48,266,360	46,650,024	6,997,503,600	97.76%	94.50%	45.19%
	b3	51,311,878	49,811,402	7,471,710,300	97.71%	94.40%	45.39%
Leaf	L1	48,972,934	46,554,096	6,983,114,400	97.98%	95.01%	45.75%
	L2	49,417,890	46,956,672	7,043,500,800	98.12%	95.30%	46.12%
	L3	50,353,228	47,904,954	7,185,743,100	97.98%	94.98%	46.66%
flower stalk	stk1	47,707,116	46,109,686	6,916,452,900	97.56%	94.82%	44.97%
	stk2	49,266,326	47,617,650	7,142,647,500	97.58%	94.10%	44.94%
	stk3	49,616,790	47,901,748	7,185,262,200	97.65%	94.15%	45.05%
flower scape	sc1	51,088,588	49,381,988	7,407,298,200	97.71%	94.01%	45.47%
	sc3	48,161,674	46,494,284	6,974,142,600	97.73%	94.41%	45.97%
	sc4	49,055,630	47,404,402	7,110,660,300	97.56%	94.42%	46.08%
pistil	pi1	49,512,126	47,904,954	7,185,743,100	97.60%	94.19%	45.86%
	pi2	48,241,606	46,650,024	6,997,503,600	97.44%	93.85%	45.61%
	pi4	48,188,664	46,554,096	6,983,114,400	97.66%	94.37%	46.05%
petal	pe1	48,803,722	47,210,776	7,081,616,400	97.60%	94.18%	44.99%
	pe3	48,089,276	46,592,162	6,988,824,300	97.71%	94.98%	45.31%
	pe4	48,400,432	46,554,096	6,983,114,400	97.96%	94.98%	45.49%
stamen	stn1	49,577,780	47,908,688	7,186,303,200	97.55%	94.28%	46.02%
	stn2	47,879,494	46,226,436	6,933,965,400	97.46%	94.05%	46.51%
	stn4	51,144,270	49,434,136	7,415,120,400	96.73%	93.90%	46.57%
seed	seed1	49,891,584	47,599,074	7,139,861,100	97.91%	94.11%	47.11%
	seed2	48,633,158	46,353,526	6,953,028,900	97.90%	94.87%	46.66%
	seed4	48,412,552	46,542,152	6,981,322,800	97.86%	94.85%	45.25%
Total		1,325,683,066	1,277,585,202	191.64 G			

Supplementary Table S8 The expression pattern of each unigene based on FPKM value.

In the attachment

Supplementary Table S9 List of *LauCYP96T* homolog genes cloned from full-length transcriptome sequencing data.

mRNA_id	% identity to NpsCYP96T1 (nt)	% identity to NpsCYP96T1 (aa)	% identity to Nt êCYP96T6 (aa)	Annotation	Gene name
HQ_transcript_26411	81.39	73.10	69.98	CYP96 family	LauCYP96T1-like
HQ_transcript_29398	81.32	72.51	69.79	CYP96 family	LauCYP96T1-like
HQ_transcript_33482	92.94	90.93	85.14	CYP96 family	LauCYP96T1
HQ_transcript_35530	81.45	73.88	71.15	CYP96 family	LauCYP96T1-like
HQ_transcript_42692	80.88	64.91	61.21	CYP96 family	LauCYP96T1(partial)
CYP96T1-clone1	93.13	91.81	85.96	CYP96 family	LauCYP96T1
CYP96T1-clone2	81.00	72.90	70.37	CYP96 family	LauCYP96T1-like-1
CYP96T1-clone3	81.65	73.68	71.15	CYP96 family	LauCYP96T1-like-2
CYP96T1-clone4	81.52	74.07	71.15	CYP96 family	LauCYP96T1-like-3

Supplementary Table S10 List of annotation of *CYP96T* homolog genes in different plant species.

name	Length (bp/aa)	Species	accession number (nucleotide sequences)	accession number (protein sequences)	References
NpsCYP96T1	1542/513	*Narcissus* sp. *aff. pseudonarcissus*	KT693311	AMO65741.1/ A0A140IL90.1	Kilgore et al., 2016
NpsCYP96T2	1542/513	*Narcissus* sp. *aff. pseudonarcissus*	KT693312	AMO65742.1/A0A140IL91.1	Kilgore et al., 2016
NpsCYP96T3	1542/513	*Narcissus* sp. *aff. pseudonarcissus*	KT693313	AMO65743.1/A0A140IL92.1	Kilgore et al., 2016
NtêCYP96T1	1542/513	*Narcissus* cv. Tête-à-Tête	-	-	Mehta et al., 2023
NtêCYP96T6	1542/513	*Narcissus* cv. Tête-à-Tête	-	-	Mehta et al., 2023
CpoCYP96T1	1638/545	*Crinum x powellii cultivar Phoenix*	OQ923373	WGU11344.1	
CpoCYP96T2	1254/417	*Crinum x powellii cultivar Phoenix*	OQ923375	WGU11346.1	
LloCYP96T1	1539/512	*Lycoris longiuba*	c146899_g2		Li et al., 2020
LloCYP96T1-1	1557/518	*Lycoris longiuba*	-	-	Mehta et al., 2023 (re-analyze)
NpaCYP96T1	1605/534	*Narcissus papyraceus*	MF979870	MF979870	Hotchandani et al., 2019
NpaCYP96T2	1605/534	*Narcissus papyraceus*	MF979871	AXU39907.1	Hotchandani et al., 2019
LaeCYP96T1-partial	1602/533	*Leucojum aestivum*	MW971979	UIP35232.1	Tousignant et al., 2022 (partial)
LaeCYP96T2-partial	1602/533	*Leucojum aestivum*	MW971980	UIP35233.1	Tousignant et al., 2022 (partial)
NpsKingAlfred_CYP96T1	1542/513	*Narcissus pseudonarcissus 'King Alfred'*	MF416097	AUG71942.1	Singh and Desgagné-Penix, 2017
NpsKingAlfred_CYP96T2	1542/513	*Narcissus pseudonarcissus 'King Alfred'*	MF416098	AUG71943.1	Singh and Desgagné-Penix, 2017
NpsKingAlfred_CYP96T6-1	1290/429	*Narcissus pseudonarcissus 'King Alfred'*	-	-	Mehta et al., 2023 (re-analyze; partial)

Supplementary Table S11 Sequences of LauCYP96T chimeras and mutants.

In the attachment

Figure S1. Multiple nucleotide acid sequences alignments between *NpsCYP96T1* and *CYP96T1*-like sequences in *L. aurea* full-length transcriptome. Arrows indicate specific primer sequences used for *CYP96T1*-like gene cloning.

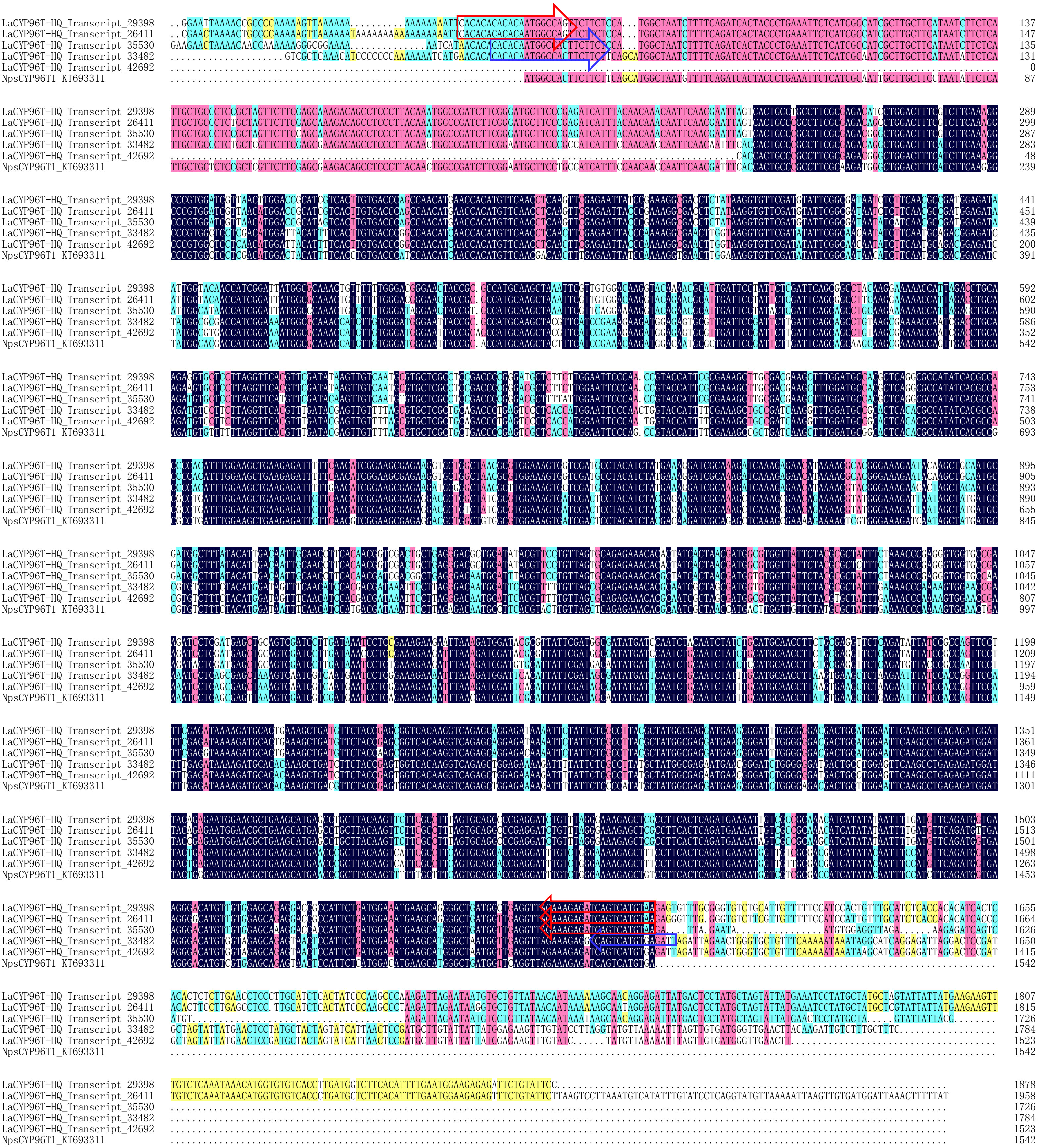

Figure S2. Co-expression analysis of annotated cytochrome P450 genes in different tissues as well as under MJ treatment at different time.

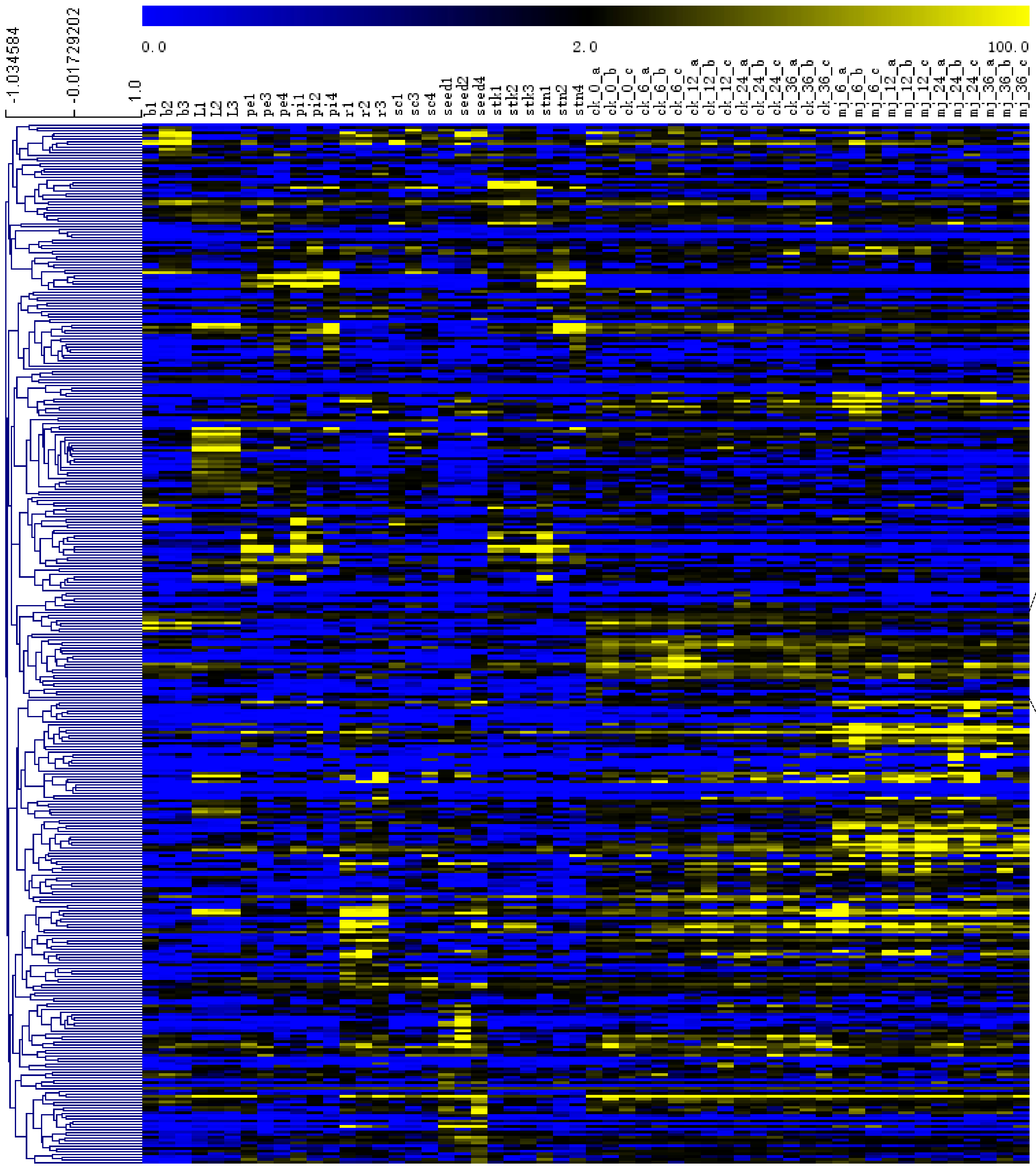

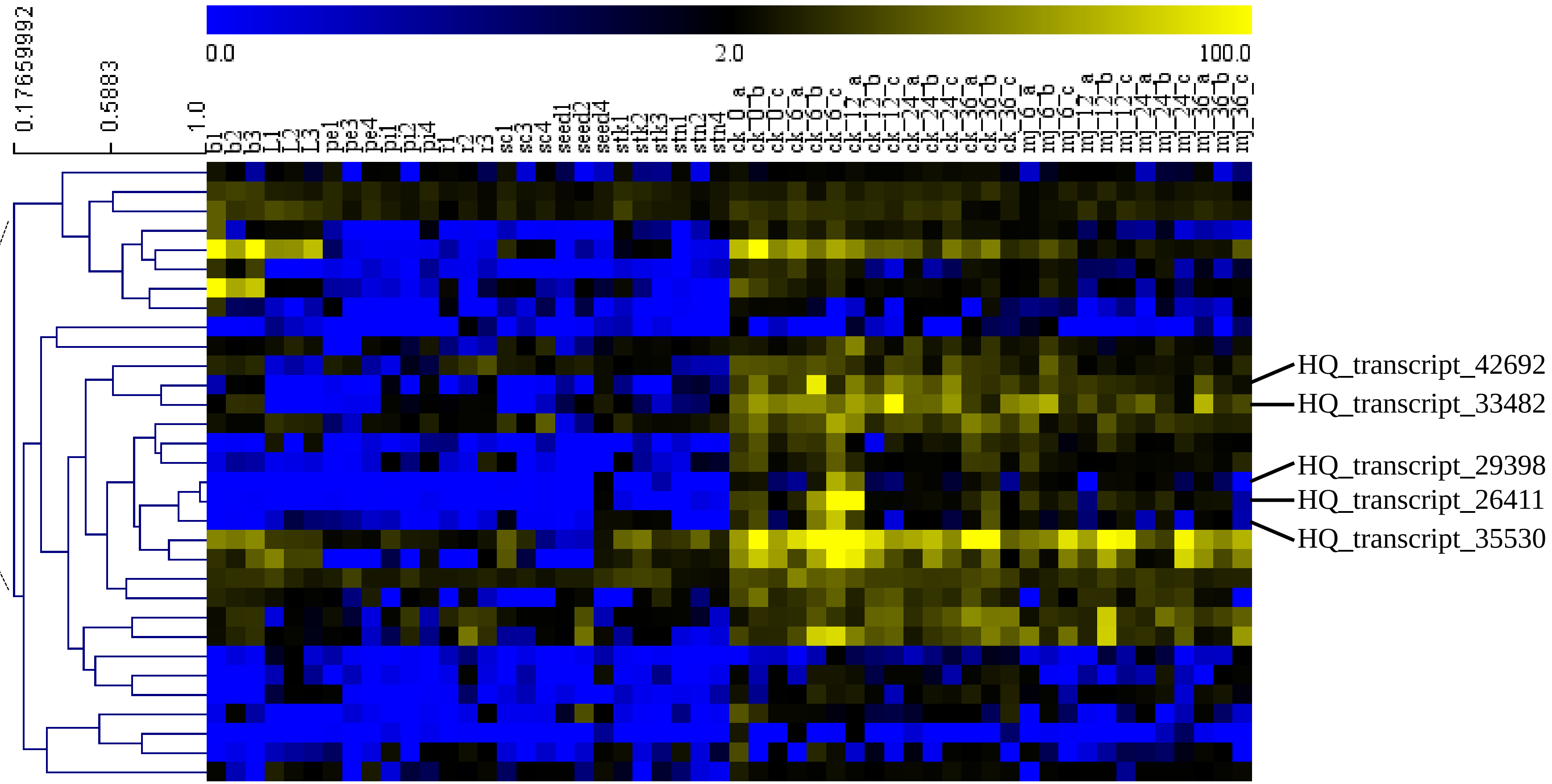

Figure S3. Phylogenetic trees of representative CYP96T1-like sequences in *L. aurea* transcriptome, *LauCYP96T* cloned by RT-PCR, and *CYP96T* from plant species. The nucleotide acid (A) and amino acid (B) were used for alignments with ClustalW, and tree-building was constructed in MEGA version 5.2 using the maximum-likelihood method with 1000 replicate bootstrap support. The characterized CYP96T from *Narcissus* sp. *aff. pseudonarcissus* (NpsCYP96T), *Narcissus pseudonarcissus* var. King Alfred (NpsKingAlfred_CYP96T), *Narcissus* cv. Tête-à-Tête (NtêCYP96T), *Crinum x powellii cultivar Phoenix* (CpoCYP96T), *Narcissus papyraceus* (NpaCYP96T), *Leucojum aestivum* (LaeCYP96T), and CYP96T from *Lycoris longituba* (LloCYP96T) were listed. Numbers at the nodes indicate the percent bootstrap values. The bar at bottom shows 0.02 nucleotide acid and 0.05 amino acid substitution, respectively.

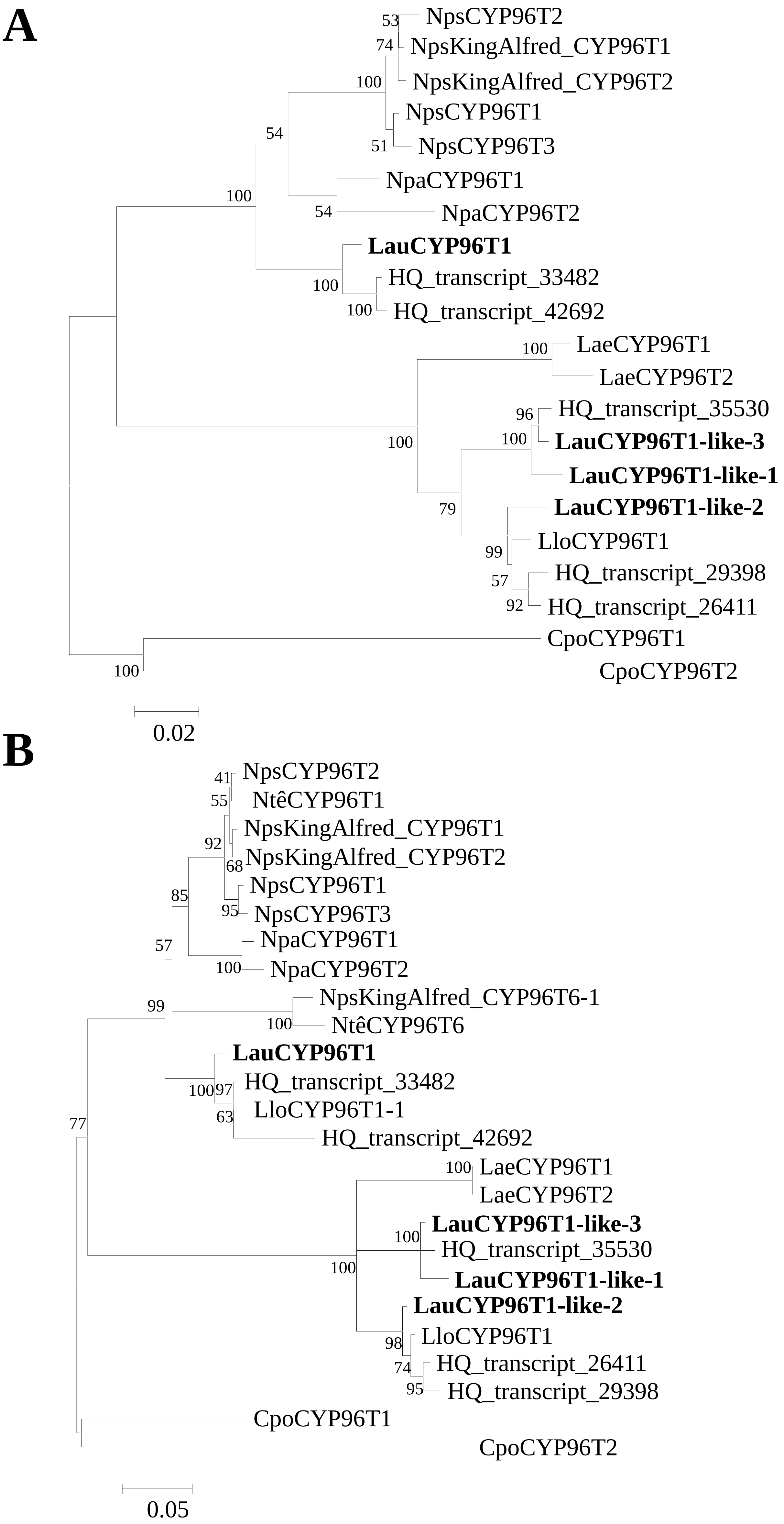
A
NpsCYP96T2
NpsKingAlfred_CYP96T1
NpsKingAlfred_CYP96T2
NpsCYP96T1
NpsCYP96T3
NpaCYP96T1
NpaCYP96T2
LauCYP96T1
HQ_transcript_33482
HQ_transcript_42692
LaeCYP96T1
LaeCYP96T2
HQ_transcript_35530
LauCYP96T1-like-3
LauCYP96T1-like-1
LauCYP96T1-like-2
LloCYP96T1
HQ_transcript_29398
HQ_transcript_26411
CpoCYP96T1
CpoCYP96T2
0.02
B
NpsCYP96T2
NtêCYP96T1
NpsKingAlfred_CYP96T1
NpsKingAlfred_CYP96T2
NpsCYP96T1
NpsCYP96T3
NpaCYP96T1
NpaCYP96T2
NpsKingAlfred_CYP96T6-1
NtêCYP96T6
LauCYP96T1
HQ_transcript_33482
LloCYP96T1-1
HQ_transcript_42692
LaeCYP96T1
LaeCYP96T2
LauCYP96T1-like-3
HQ_transcript_35530
LauCYP96T1-like-1
LauCYP96T1-like-2
LloCYP96T1
HQ_transcript_26411
HQ_transcript_29398
CpoCYP96T1
CpoCYP96T2
0.05

Figure S4. ^{1}H-NMR spectrum of synthetic 4’-*O*-methylnorbelladine.

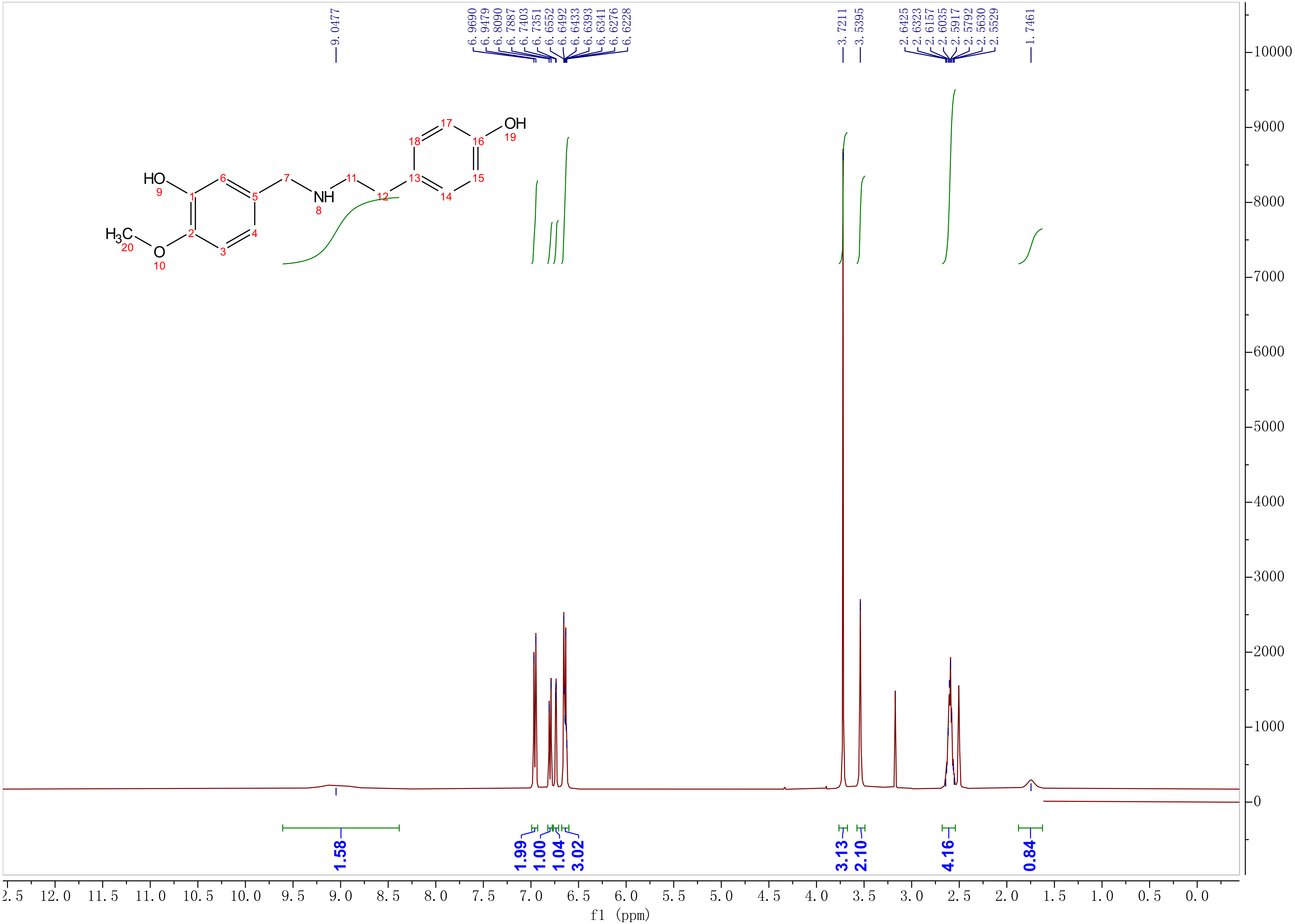

Figure S5.^{1}H-NMR spectrum of synthetic noroxomaritidine.

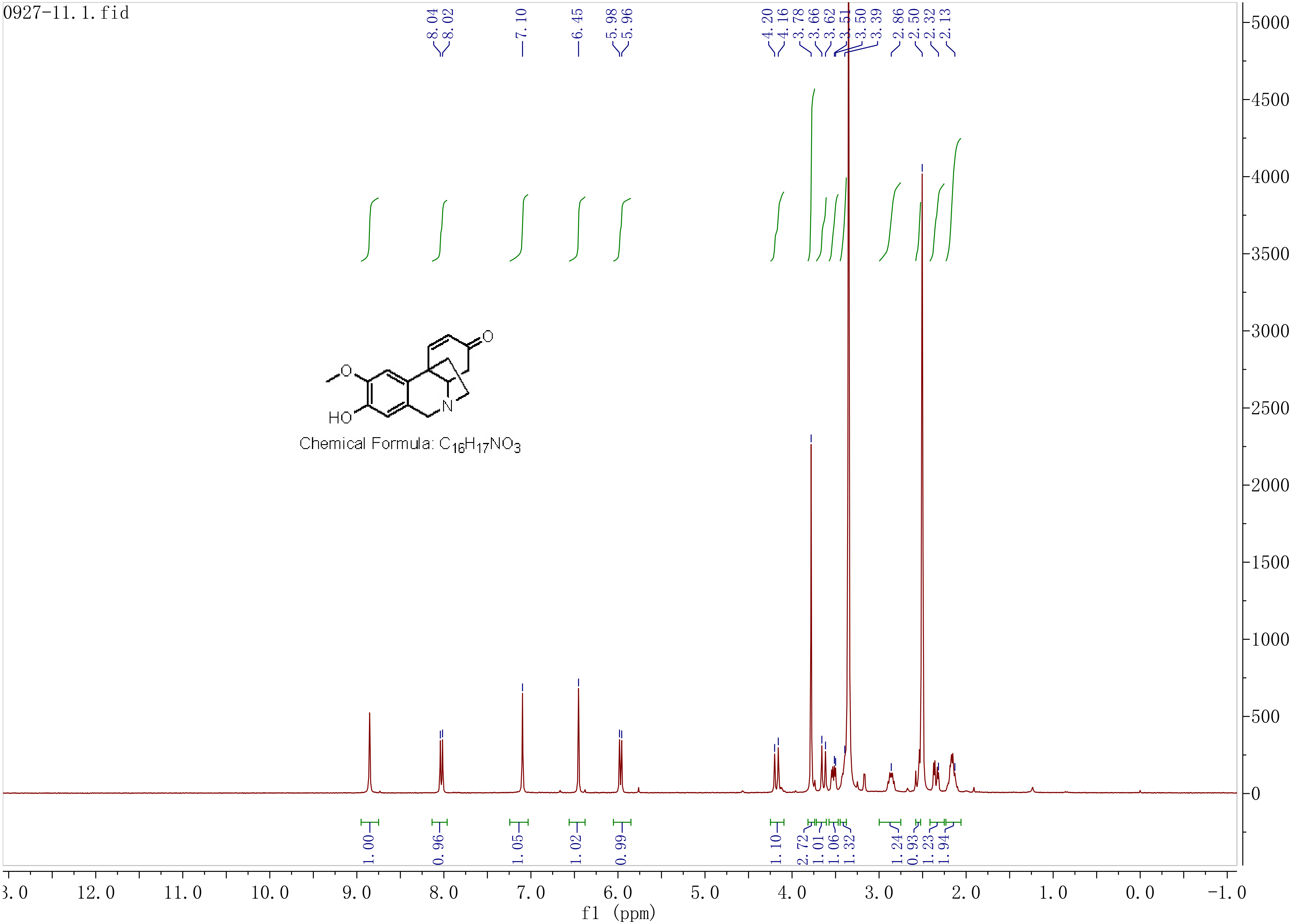

Figure S6. ^{1}H-NMR spectrum of synthetic demethylnarwedine.

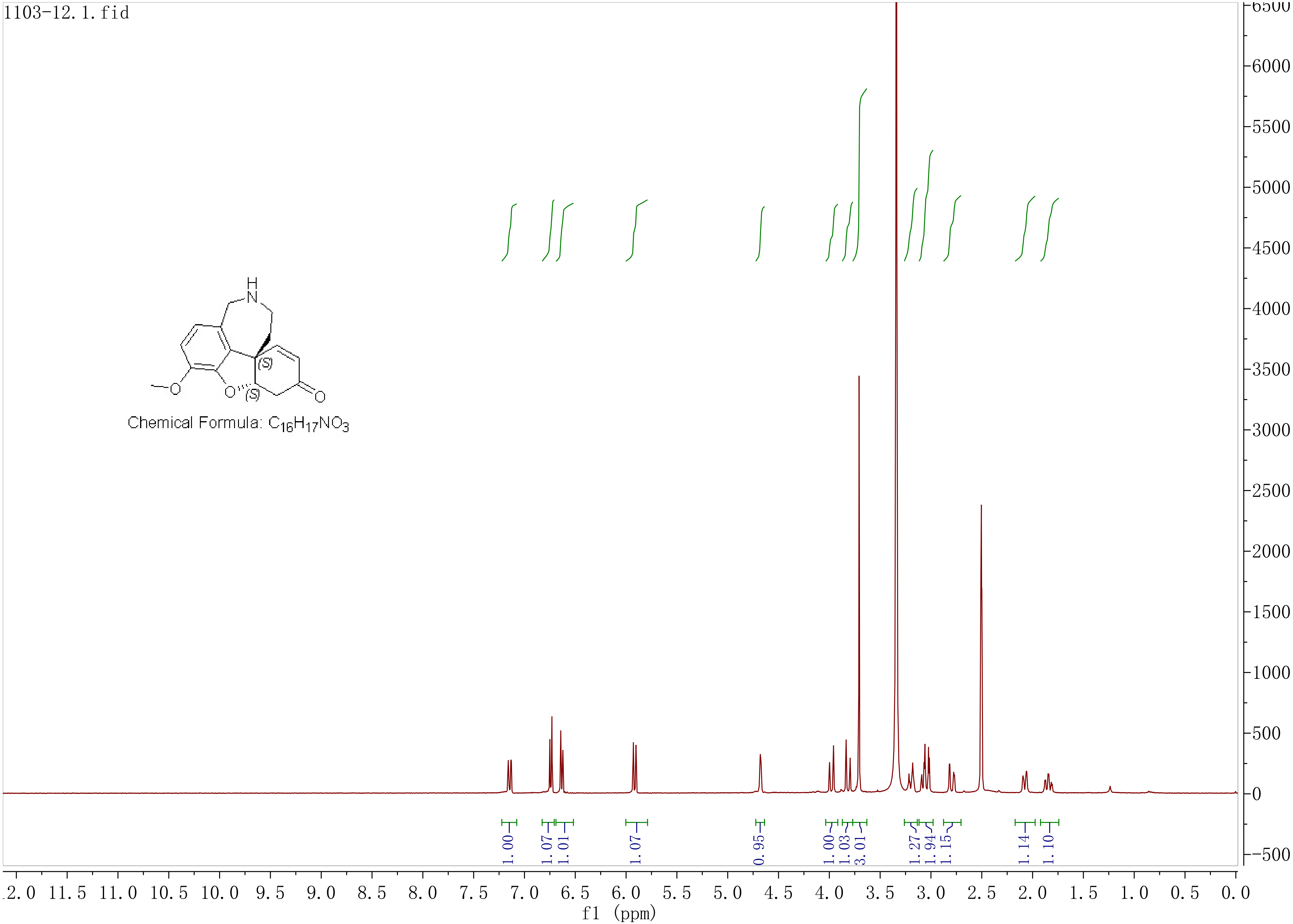

Figure S7. Mass spectra of the products catalyzed by LauCYP96T1 and LauCYP96T1-like proteins comparing to each authentic standard.

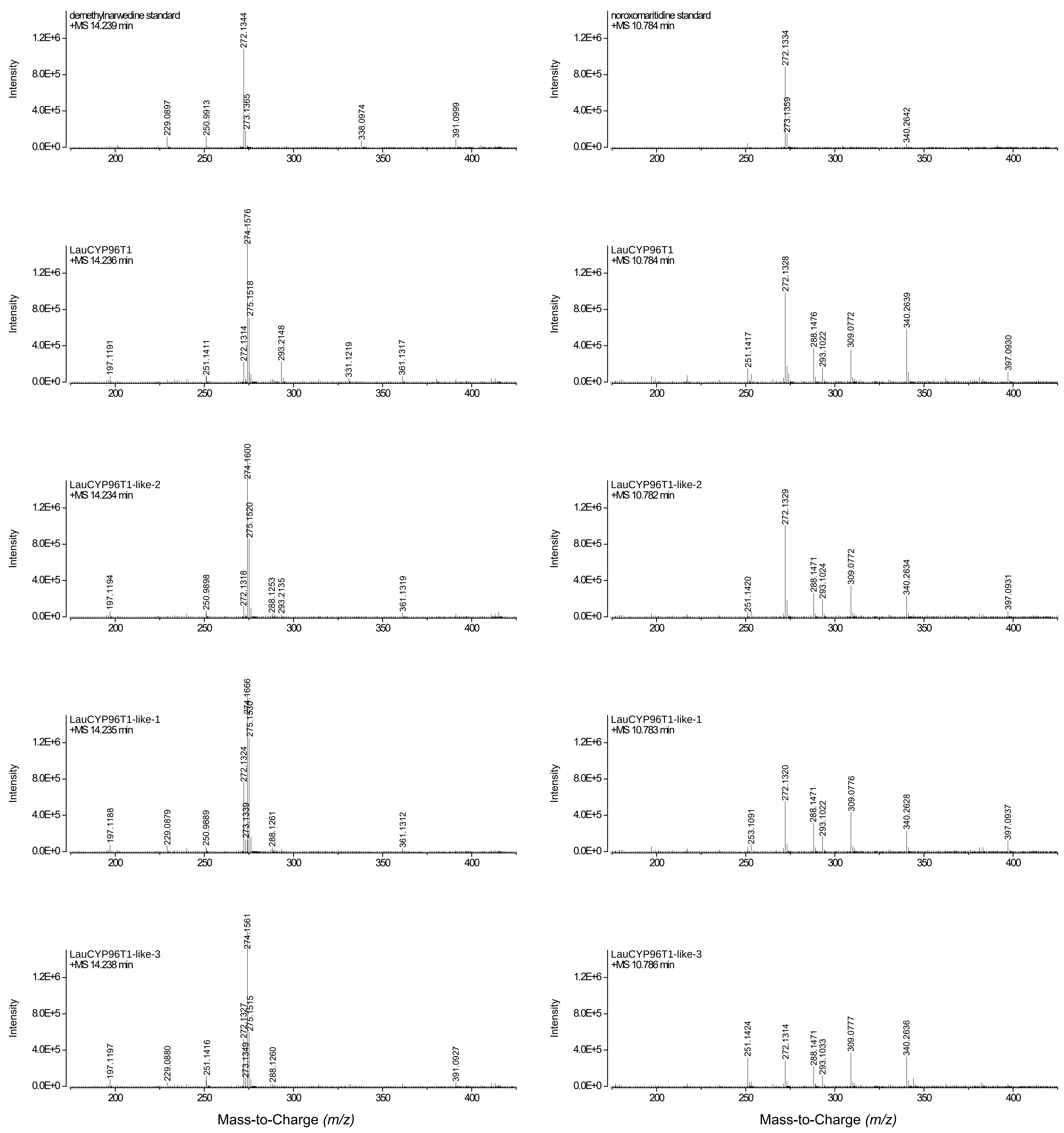

Figure S8. Homology modeling of LauCYP96T1, LauCYP96T1-like-1, LauCYP96T1-like-2, and LauCYP96T1-like-3.

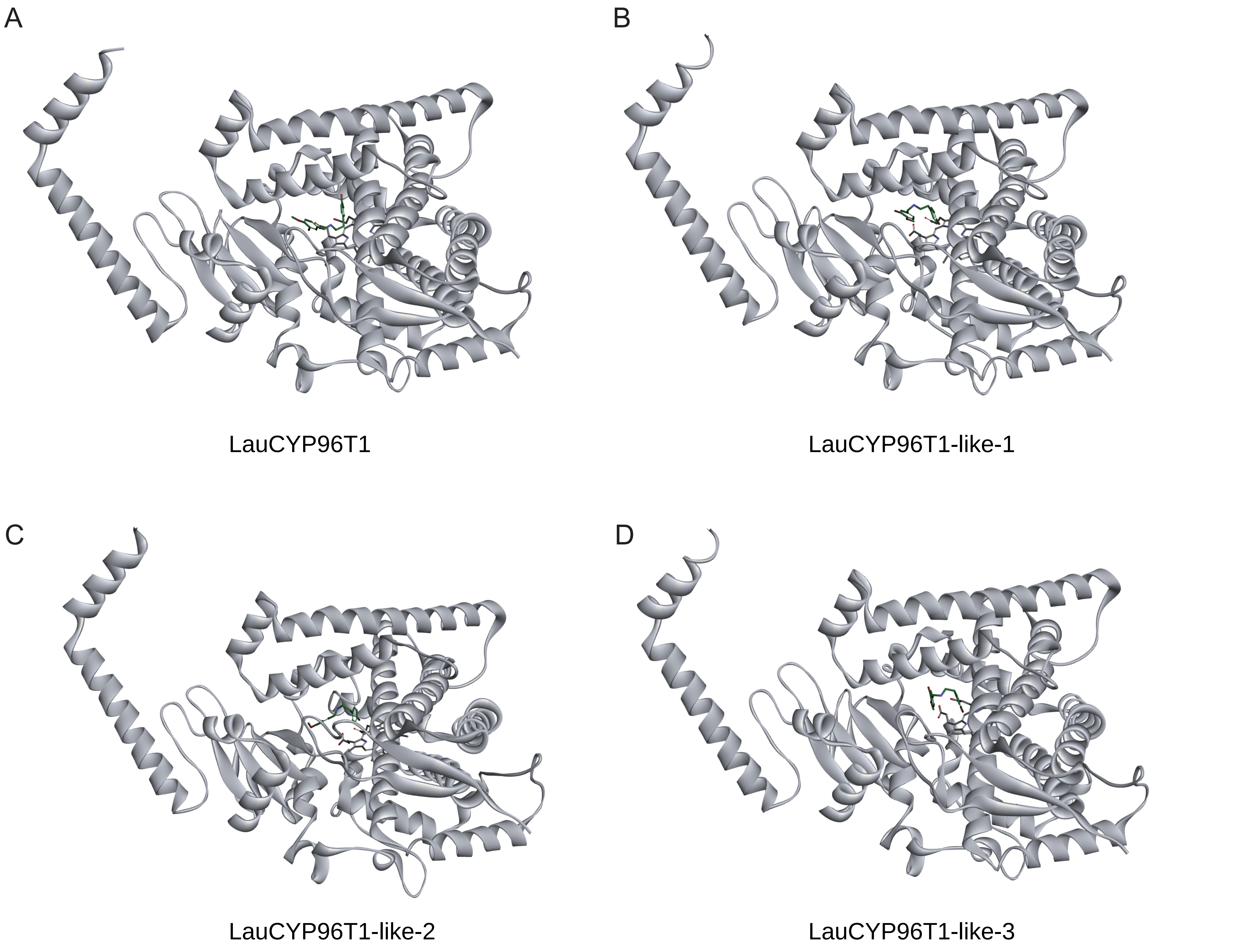

References:

Hotchandani, T., de Villers, J., Desgagné-Penix, I. (2019). Developmental regulation of the expression of Amaryllidaceae alkaloid biosynthetic genes in *Narcissus papyraceus*. Genes 10, 597. https://doi.org/10.3390/genes10080594

Kilgore, M. B., Augustin, M. M., May, G. D., Crow, J. A., and Kutchan, T. M. (2016b). CYP96T1 of *Narcissus* sp. aff. *pseudonarcissus* catalyzes formation of the *para-para'* C–C phenol couple in the Amaryllidaceae alkaloids. Front. Plant Sci. 7, 225. https://doi.org/10.3389/fpls.2016.00225.

Li, Q., Xu, J., Yang, L., Zhou, X., Cai, Y., Zhang, Y. (2020). Transcriptome analysis of different tissues reveals key genes assocated with galanthamine biosynthesis in *Lycoris longituba*. Front. Plant Sci. 11, 519752. https://doi.org/10.3389/fpls.2020.519752

Mehta, N., Meng, Y., Zare, R., Kamenetsky-Goldstein, R. and Sattely, E. (2023). A developmental gradient reveals biosynthetic pathways to eukaryotic toxins in monocot geophytes. BioRxiv https://doi.org/10.1101/2023.05.12.540595.

Singh, A., Desgagné-Penix, I. (2022). Transcriptome and metabolome profiling of *Narcissus pseudonarcissus* 'King Alfred' reveal components of Amaryllidaceae alkaloid metabolism. Sci. Rep. 7, 17356. https://doi.org/10.1038/s41598-017-17724-0

Tousignant, L., Diaz-Garza, A. M., Majhi, B. B., Gélinas, S. E., Singh, A., Desgagné-Penix, I. (2022). Transcriptome analysis of *Leucojum aestivum* and identification of genes involved in norbelladine biosynthesis. Planta 255, 30. https://doi.org/10.1007/s00425-021-03741-x
